# Supplementary material for: OVOL1 Influences the Determination and Expansion of iPSC Reprogramming Intermediates
Source: Stem Cell Reports. 2019 Jan 10;12(2):319–32. doi: 10.1016/j.stemcr.2018.12.008 (PMC6372973; doi:10.1016/j.stemcr.2018.12.008)
Supplement: Document S1. Supplemental Experimental Procedures and Figures S1–S5 [file mmc1.pdf]

Stem Cell Reports, Volume *12*

Supplemental Information

OVOL1 Influences the Determination and Expansion of iPSC Reprogramming Intermediates

Harunobu Kagawa, Ren Shimamoto, Shin-Il Kim, Fabian Oceguera-Yanez, Takuya Yamamoto, Timm Schroeder, and Knut Woltjen

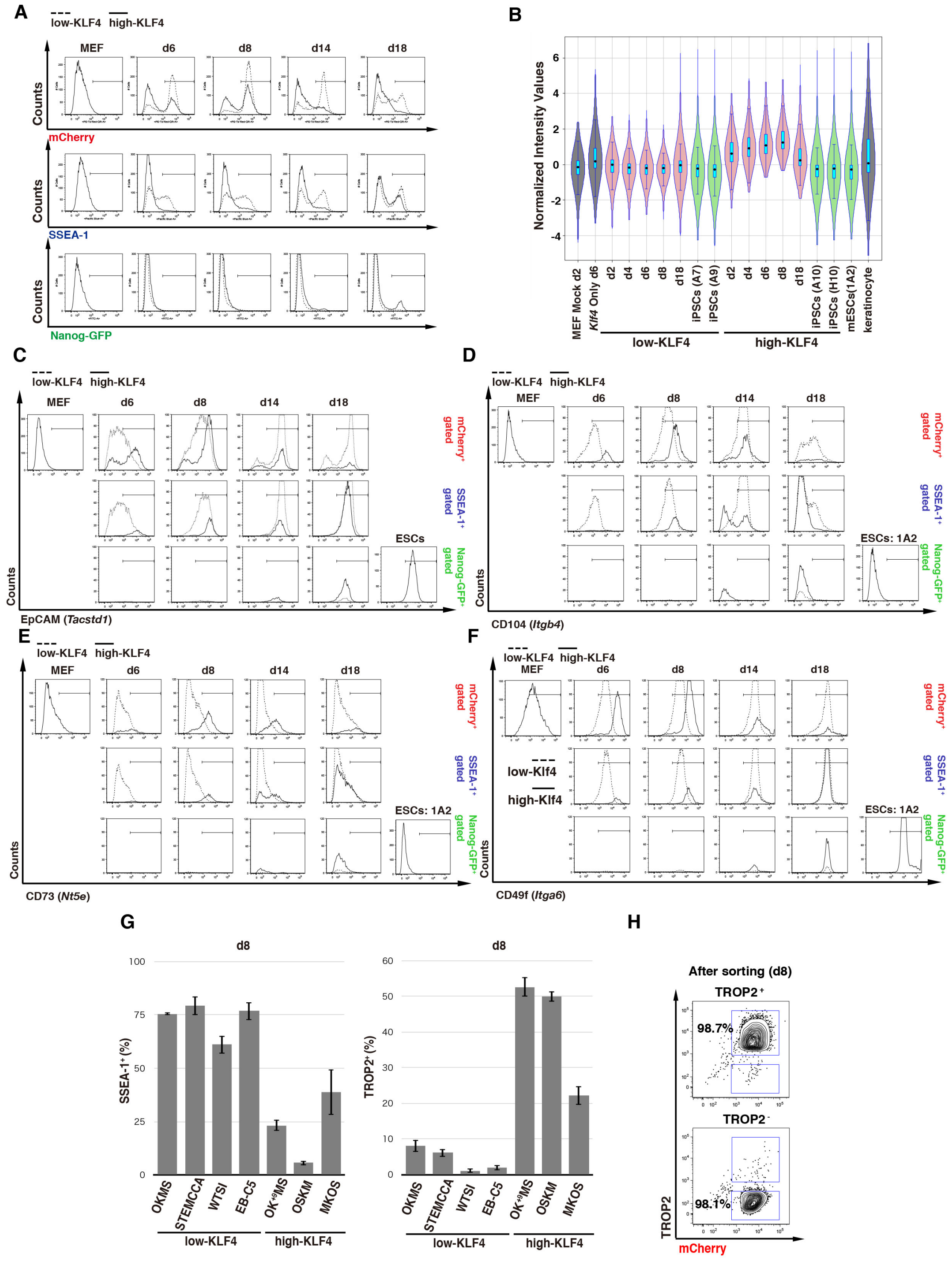

Figure S1. Characterizing low- and high-KLF4 mediated reprogramming

(A) Flow cytometry analysis for mCherry, SSEA-1, and Nanog-GFP during low- and high-KLF4 MEF reprogramming.

(B) Violin plot of normalized intensity values for high-KLF4 specific genes in low- or high-KLF4 reprogramming processes. The box plot displays the median, 25th, and 75th percentiles.

(C-F) FACS analysis of cell surface marker dynamics. Histograms are grouped by analysis day (columns) or population gating (rows). Dashed lines and straight lines represent low-KLF4 and high-KLF4, respectively.

(G) Proportion of SSEA-1^{+} (left) and TROP2^{+} (right) on d8 in publically available low- and high-KLF4 reprogramming systems (Low: OKMS, STEMCCA, WTSI and EB-C5; High: OK^{+9}MS, OSKM and MKOS). Means ± SD for three independent experiments.

(H) FACS plots verifying sorting efficiency for TROP2^{-} and TROP2^{+} populations at d8 in high-KLF4.

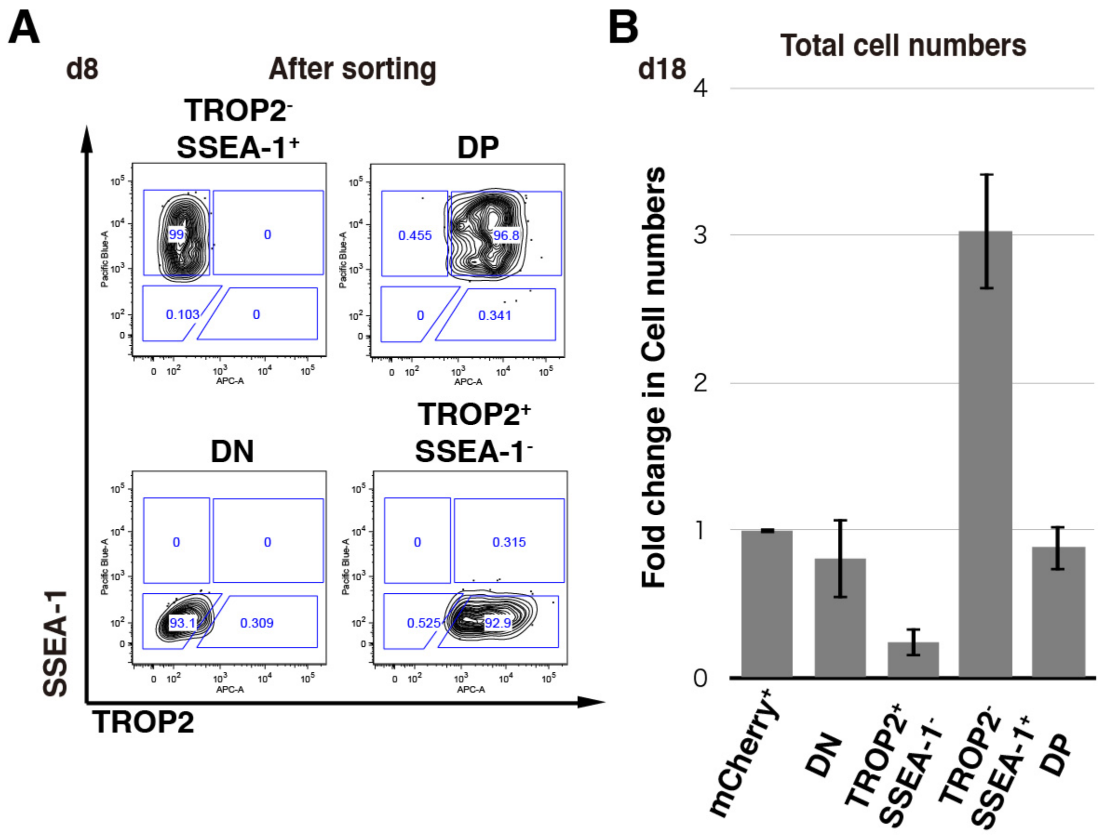

Figure S2. Reprogramming capacity of TROP2 and SSEA-1 marked populations

(A) FACS plots verifying sorting efficiency for double negative (DN), TROP2$^+$SSEA-1$^-$, TROP2$^-$SSEA-1$^+$, and double positive (DP) populations at d8 in high-KLF4.

(B) Total cell numbers on d18 following d8 sorting based on TROP2 and SSEA-1 presentation. Total cell numbers are normalized to mCherry$^+$. Means ± SD for three independent experiments (n=3).

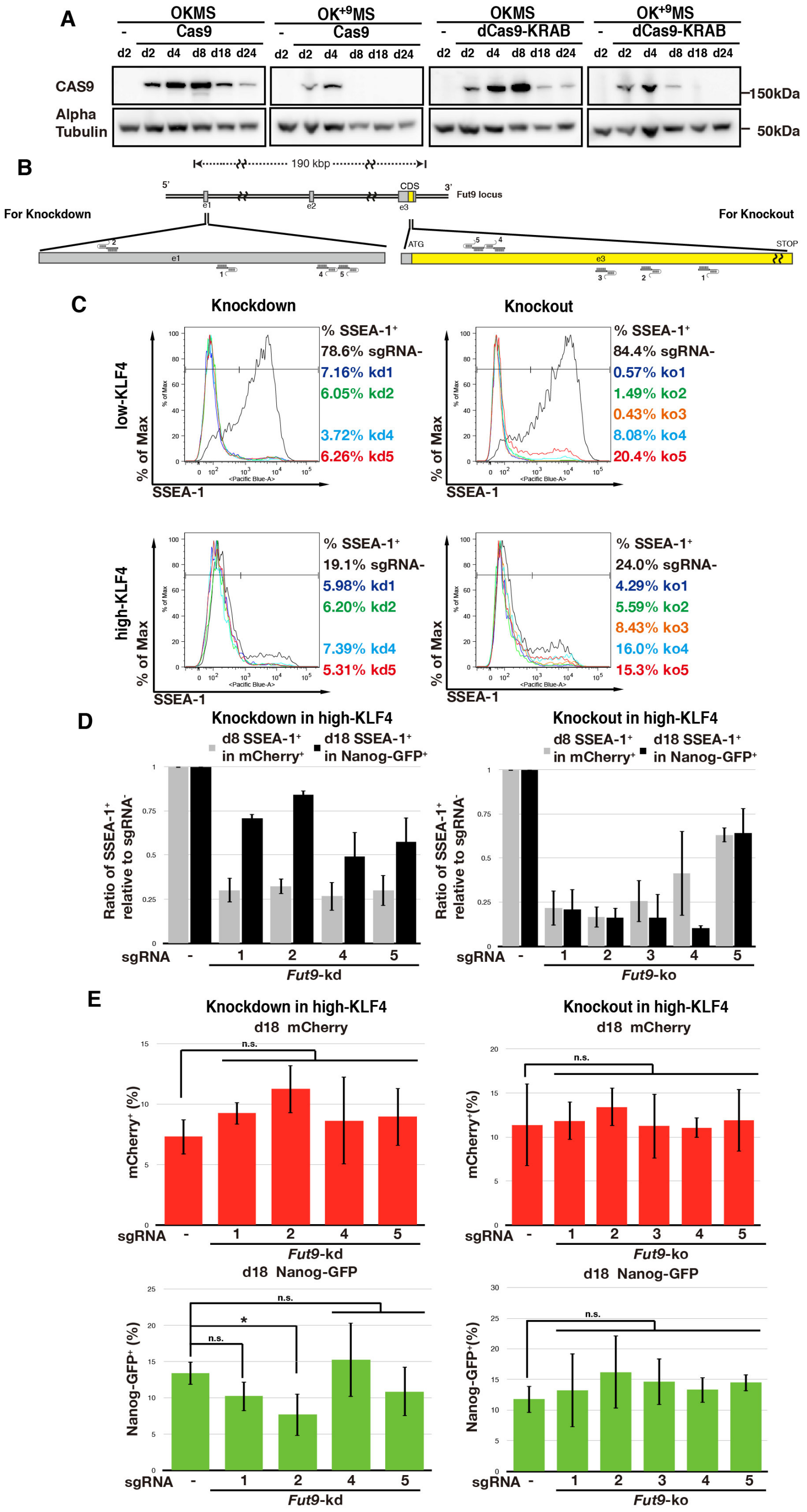

Figure S3. Establishment of CRISPR/Cas9 knockdown and knockout systems during reprogramming

(A) Western blot analysis of CAS9 and KRAB-dCas9 expression in low- and high-KLF4 from d2 – d24. Alpha Tubulin was used as a loading control.

(B) Schematic diagrams of the *Fut9* locus (NC_000070.6, 25609333..25800003, complement) with detailed segments of exon 1 and exon 3 to show sgRNA target sites.

(C) *Fut9* knockdown (left) and knockout (right) efficiencies by flow cytometry. Histograms depict the SSEA-1 presentation at d8 in low-KLF4 (top) and high-KLF4 (bottom).

(D) SSEA-1 presentation following *Fut9* knockdown (left) and knockout (right) in high-KLF4 detected by flow cytometry at d8 in the mCherry$^+$ population and at d18 in the Nanog-GFP$^+$ population. Bar graphs depict the normalized percentage of SSEA-1$^+$ population to sgRNA-. Means ± SD for three independent experiments.

(E) Proportion of mCherry$^+$ (top) and Nanog-GFP$^+$ (bottom) on d18 following *Fut9* knockdown (left) and knockout (right) in high-KLF4. Only *Fut9*-kd2 presented a sigfinificant difference in acquisition of Nanog-GFP. Means ± SD for three independent experiments. *, $p<0.05$. Student t-test

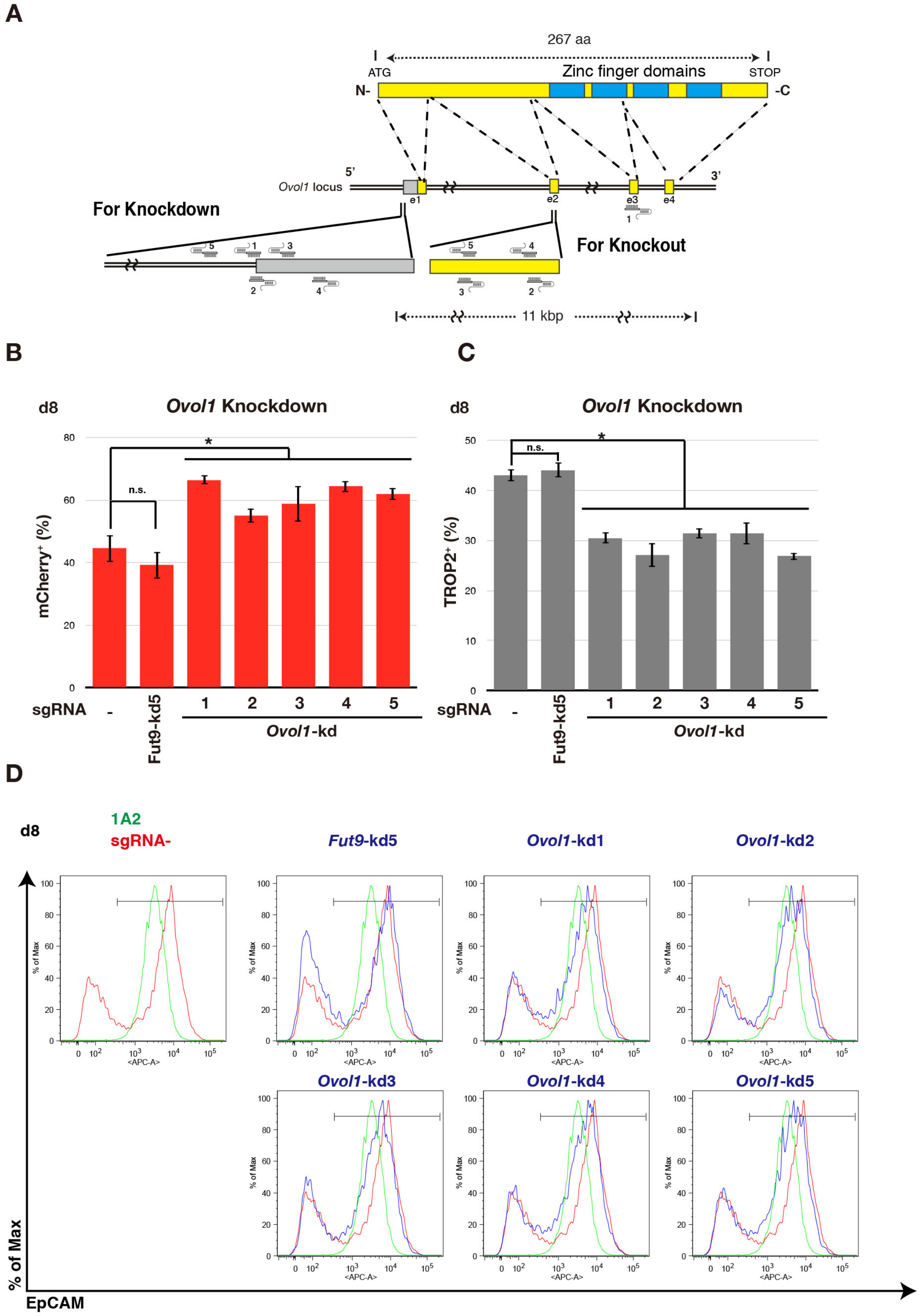

Figure S4. Cas9 and KRAB-Cas9 sgRNA design and validation for *Ovol1*

(A) Schematic diagram showing the *Ovol1* locus (NC_000085.6, 5549137..5560575, complement) with detailed segments of exon 1 and exon 2 to show sgRNA target sites. The OVOL1 protein (UniProt: Q9WTJ2), including the zinc finger domains, is shown above.

(B) Proportion of $mCherry^+$ on d8 in high-KLF4 reprogramming with or without *Ovol1* knockdown. Means ± SD for five independent experiments. *, $p<0.05$. Student t-test

(C) Proportion of $TROP2^+$ on d8 in high-KLF4 reprogramming with or without *Ovol1* knockdown. Means ± SD for three independent experiments. *, $p<0.05$. Student t-test

(D) FACS analysis of EpCAM expression dynamics. Each knockdown sample is presented by blue lines. Green lines and red lines represent 1A2 and sgRNA-, respectively.

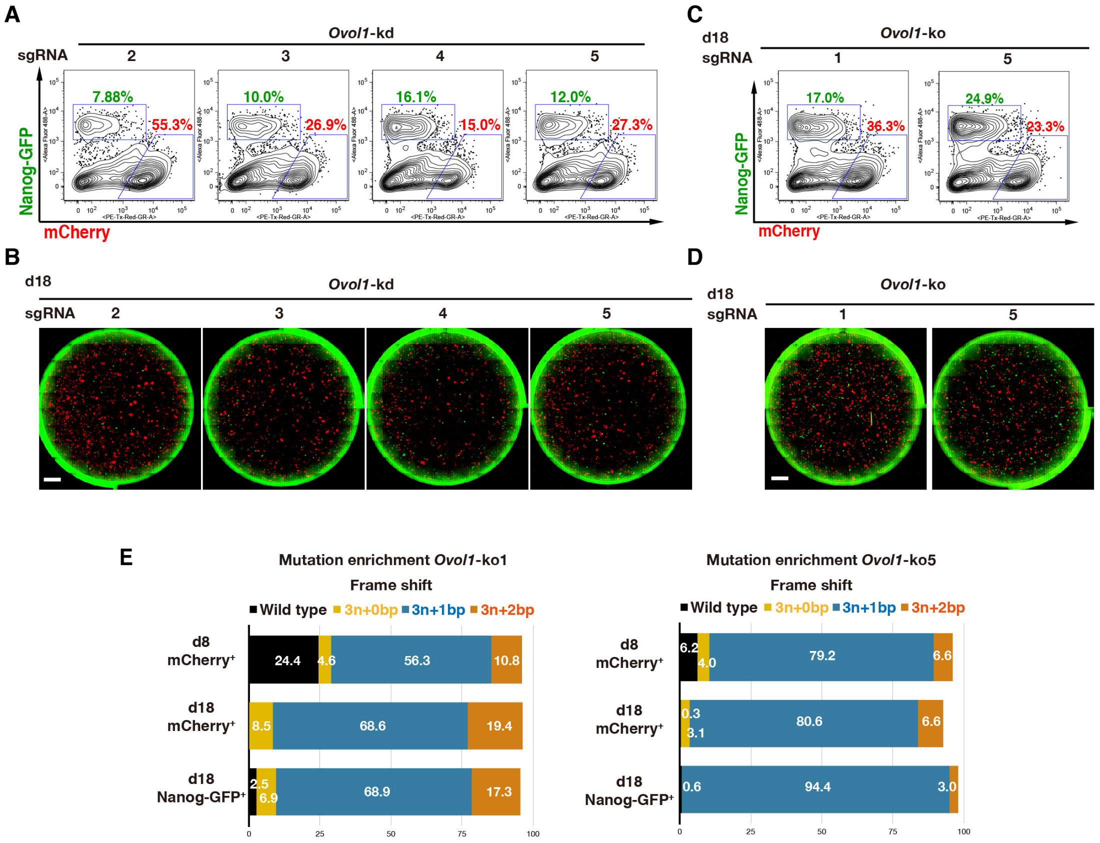

Figure S5. Effects of *Ovol1* knockdown or knockout on reprogramming efficiency

(A) FACS analysis of Nanog-GFP and mCherry on d18 following *Ovol1* knockdown in high-KLF4.

(B) Whole-well fluorescence microscopy images for Nanog-GFP and mCherry on d18 following *Ovol1* knockdown in high-KLF4. Scale bars, 4,000 μm.

(C) FACS analysis of Nanog-GFP and mCherry on d18 following *Ovol1* knockout in high-KLF4.

(D) Whole-well fluorescence microscopy images for Nanog-GFP and mCherry on d18 following *Ovol1* knockout in high-KLF4. Scale bars, 4,000 μm.

(E) Distribution of mutation frequencies predicted by TIDE in d8 mCherry$^+$, d18 mCherry$^+$ and d18 Nanog-GFP$^+$ populations following *Ovol1* knockout in high-KLF4. Mutant alleles were categorized based on a resulting in-frame indels 3n + 0 bp (yellow), frame-shifted indels 3n + 1 bp (blue), or 3n + 2 bp (orange). Wild type (black) alleles showed no indel. Data are representative of two independent experiments.

Supplemental Experimental Procedures

Plasmid Construction

A list of sequence-verified plasmids and primers used for cloning is listed in Table S4. Complete sequences are available through Addgene (plasmid #'s 120352-120360) or upon request. Detailed cloning histories are available upon request as Snapgene files. Briefly, PB-TAC-Cas9 plasmid were generated by standard cloning using restriction digestion and ligation. The Cas9 cassette was cloned from pX330-U6-Chimeric_BB-CBh-hSpCas9 provided by Feng Zhang (Addgene plasmid # 42230). The plasmid PB-TAC-KRAB-dCas9 was generated by PCR amplification, restriction digestion and ligation. KRAB-dCas9 was cloned from pUCM-AAV-NC-TO-KC provided by Bruce Conklin (Mandegar et al., 2016). Yamanaka factors were inserted into these plasmids by restriction digestion and ligation or by Gateway LR clonase reaction (Invitrogen, Cat.11791100) as described previously (Woltjen et al., 2009). For sgRNA expression, PB-U6 plasmid was generated by restriction digestion and ligation. The sgRNA scaffold was cloned from pX330-U6-Chimeric_BB-CBh-hSpCas9. Oligos encoding sgRNA were annealed and cloned into PB-U6 linearized with BbsI as previously described (Ran et al., 2013). The resulting plasmids were sequence verified using primer dna790. sgRNA identification numbers (ie. kd1-5 and ko1-5) are ordered based on the predicted activity score from previously published sgRNA libraries (Horlbeck et al., 2016; Koike-Yusa et al., 2014). For complementation experiments, a synthesized DNA fragment composed of the *Ovol1* cDNA sequence flanked by attB1 and attB2 sites (IDT) was cloned into pDONR221 using Gateway BP clonase reaction (Invitrogen Cat.11789020). PB-TAB vectors containing *Ovol1* or *LacZ* (mock) were generated by Gateway LR clonase reaction.

MEF Isolation and PB Reprogramming

MEFs were isolated from E13.5 mouse embryos resulting from the mating of homozygous Nanog-GFP (Okita et al., 2007) transgenic males and homozygous ROSA26-rtTA (Ohnishi et al., 2014) transgenic females on a C57BL/6 background, or wild-type C57BL/6 mice (without ROSA26-rtTA or Nanog-GFP transgenes), and cultured as described previously (Woltjen et al., 2016). Animal care and experiments using animal tissues and primary cell culture were approved by the CiRA Animal Experiment Committee in accordance with Kyoto University guidelines. MEFs were seeded in DMEM containing 10% FBS, penicillin-streptomycin, and L-glutamine on gelatin coated 6-well dishes at a density of 1×10^5 cells per well. After a 24 hr culture, FugeneHD (Promega, Cat.E2312) was used to transfect cells at a Fugene/DNA ratio of 4:1. Different amounts of transposons were utilized in transfection to achieve similar transfection efficiencies based on mCherry induction (500 ng: PB-TAC-OKMS and -OK^{+9}MS, PB-U6-sgRNA, PB-TAB-*LacZ*, and -*Ovol1*, 1,500 ng: PB-TAC-OKMS-Cas9, -OKMS-KRAB-dCas9, OK^{+9}MS-Cas9 and -OK^{+9}MS-KRAB-dCas9). 1,000 ng of pCyL43 PB transposase plasmid was used regardless of the total amount of transposons. After 24 hr, the medium was replaced with ESC medium (DMEM containing 15% FBS, penicillin-streptomycin, GlutaMAX, β-mercaptoethanol, sodium-pyruvate, non-essential amino acids, LIF, and dox [1 μg/mL]). After transfection, cells were fed daily with dox-containing ESC medium. On d8, cells were detached by using TrypLE™ Select (1×) (Thermo Fisher Scientific, Cat.12563011) and re-seeded at 3×10^5 cells

per well of gelatin coated 6-well dishes for analysis at d18. For *LacZ* and *Ovol1* complementation experiments, cells were assayed at d18 without d8 passage. For continuous culture after d8 sorting, sorted cells were re-seeded at 1×10^5 cells per well of gelatin coated 6-well dishes with 2×10^5 cells of MitomycinC treated MEFs as feeder. Total viable cell counts were performed using a TC20 Automated Cell Counter (Bio-Rad) using trypan blue exclusion.

Whole-Well Fluorescence Microscopy Imaging

Mouse fibroblasts were plated on standard tissue culture 6-well plastic plates (Greiner, Cat. 657160). Images were acquired with a Nikon BioStation CT (Nikon) equipped with GFP and mCherry fluorescence filters and phase contrast using 2× lenses. The single-plane images of each channel were stitched automatically using the automated image analysis software CL-Quant 3.0 (Nikon). Colony count analysis was performed using a custom macro for CL-Quant 3.0. Briefly, iPS colonies were initially identified using phase contrast images which served to generate an outline. Objects identified as colonies were used as regions of interest (ROI) from where the GFP and mCherry fluorescence intensities were measured. The background was subtracted from both the GFP and mCherry fluorescent images. The threshold parameters were set by adding 5 times the standard deviations to the mean intensities of GFP-negative or mCherry-negative colonies in the background subtracted images.

Flow Cytometry and Cell Sorting

For cell surface marker detection, TrypLE™ Select (1×) (Thermo Fisher Scientific, Cat.12563011) was used for cell dissociation. 3×10^5 cells were re-suspended in 100 µL of FACS buffer (PBS contained 2% of FBS) and incubated with primary antibodies and appropriate secondary antibodies on ice for 30 min each. Antibodies used in this study is described in Table S4. The samples were washed with 1 mL of FACS buffer two times after each 30 min incubation with antibodies. The samples were analyzed using a BD LSRFortessa Cell Analyzer (BD Biosciences) with BD FACSDiva software (BD Biosciences). Flow cytometry data were analyzed and generated by FlowJo software. For cell sorting, the cell population was collected on a BD FACSAria II cell sorter (BD Biosciences) following the same process as flow cytometry analysis, using MEF and mESCs as the control of the expression intensity.

Mouse epidermal keratinocytes isolation

Primary keratinocytes were isolated from newborn pups according to (Lichti et al., 2008). Briefly, mice were euthanized at post-natal day 2 by decapitation using scissors. The newborns were surgically de-limbed and subsequently washed in iodine povidone solution, 70% ethanol and finally in distilled water. The skin was removed and placed in PBS^- containing 50 µg/mL Gentamicin (Thermo Fisher Scientific, Cat. 15710-064) for 20 min. The epidermis was dissociated from the dermis by overnight treatment with Dispase II solution 0.5% w/v (Roche, Cat. 165859, 0.5U/mg) at 4°C. The epidermis was rinsed in PBS^- and cut into small pieces with a scalpel and was further incubated in neat trypsin without EDTA 0.25% (Thermo Fisher Scientific, Cat. 15050065) for 10 min at 37°C. The keratinocytes were suspended by pipetting up and down, and filtered through 70 µm Nylon mesh filters (BD Falcon). The

trypsin was blocked using one volume of D-KSFM medium containing 10% FCS and supplements (Thermo Fisher Scientific, Cat. 10744-019). After two subsequent washes, the cells were suspended in the same medium and allowed to adhere briefly onto collagen I coated dishes at 4×10^4 cells per cm^2 at 37^oC, 5% CO_2. The medium was replaced after 20 min by D-KSFM medium with 10% FCS. The next day the medium was exchanged to D-KSFM medium with supplements containing mEGF (10 µg/mL) and Cholera toxin (10^{-10} M). The keratinocytes were cultured with medium changes every two days and harvested after one week using trypsin.

Microarray Analysis

RNA isolation, data acquisition and data processing were performed as described (Kim et al., 2015). Briefly, total RNA was prepared in parallel from harvested cells using the RNeasy Mini Kit (QIAGEN) according to the manufacturer's instructions. cDNA synthesis and transcriptional amplification were performed using 200 ng of total RNA with the Whole Transcript (WT) Expression Kit (Thermo Fisher Scientific, Ambion/Affymetrix, Cat.4411973). Fragmented and biotin-labeled cDNA targets were hybridized to GeneChip Mouse Gene 1.0 ST arrays (Thermo Fisher Scientific, Affymetrix, Cat.901168) according to the manufacturer's protocols. Hybridized arrays were scanned using an Affymetrix GeneChip Scanner. Probe signal intensities were normalized with the RMA algorithm in GeneSpring. Quality, correlation, and cluster analyses were performed using GeneSpring GX software v13.1 (Agilent Technologies). The averages of two independent experiments were used for the following samples [mESCs, OKMS mCherry$^+$ intermediate reprogramming population (day 8), OK^{+9}MS mCherry$^+$ intermediate reprogramming population (day 8), primary keratinocytes, *Klf4* mCherry$^+$, and *Klf4*$^{+9}$ mCherry$^+$ intermediate reprogramming population (day 6)]. GO term analysis was performed using the NIH DAVID 6.8 Bioinformatics tool (http://david.abcc.ncifcrf.gov/). Microarray data for d6 reprogramming intermediates [LacZ population (described as MEF Mock d2), OKMS mCherry$^+$ intermediate reprogramming population (day 6), OK^{+9}MS mCherry$^+$ intermediate reprogramming population (day 6), *Klf4* mCherry$^+$ intermediate reprogramming population (day 6) and *Klf4*$^{+9}$ mCherry$^+$ intermediate reprogramming population (day 6)] were previously described and deposited in the Gene Expression Omnibus under accession number GSE65468 (Kim et al., 2015). All additional time points, including gene expression analysis for four iPSC clones previously characterized by chimera contribution (Kim et al., 2015) are available under accession number GSE116309.

qRT-PCR analysis

Total RNA were isolated from bulk reprogramming populations at d4 post-dox addition by using the RNeasy Plus Mini Kit (QIAGEN, Cat.74134) according to the manufacturer's instructions. One microgram of extracted total RNA was reverse transcribed with a SuperScript III First Strand Synthesis Kit (Thermo Fisher Scientific, Cat.18080051). qRT-PCR analysis was performed using TB Green Premix Ex Taq Ⅱ(Tli RNaseH pluse) (Takara, Cat. RR820A) and analyzed with a QuantStudio Real-Time PCR System (Life Technologies). The expression levels of target genes were normalized by the

expression level of GAPDH and calculated relative to the sgRNA(-) condition. Primer pairs for the amplification are shown in Table S4.

Protein Analysis

For Western blot analysis, cells were collected using TrypLE™ Select (1X) and washed two times using PBS before lysis. Total cell lysates were prepared by heating 1×10^6 cells to 70 °C for 10 min in 100 µL NuPAGE LDS Sample Buffer (1×) (Thermo Fisher Scientific, Cat.NP0008) containing DTT at a final concentration of 50 mM. Lysates were resolved on NuPAGE 10% Bis-Tris gels (Thermo Fisher Scientific, Cat.NP0316BOX), and probed using antibodies described in Table S4. Signals were raised using ECL Prime Western Blotting Detection Reagent (GE Healthcare, Cat.RPN2232) and detected on an ImageQuant LAS 4000 imaging system (GE Healthcare).

Immunofluorescence imaging

Cells were fixed with 4%-Paraformaldehyde Phosphate Buffer Solution (Nacalai Tesque, Inc., Cat.09154-85) for 10 min at room temperature, and masked with DPBS(-) containing 100mM glycine for 15 min at room temperature. The cells were blocked with DPBS (-) containing 3% bovine serum albumin (BSA) for 1h at room temperature. The cells were then incubated with primary antibodies described in Table S4 in DPBS (-) containing 3% BSA overnight at 4°C. After being washed in DPBS (-), the cells were stained with secondary antibodies described in Table S4 in DPBS (-) containing 1 µg/mL of DAPI (4',6-diamidino-2-phenylindole) and 3% BSA for 1h at room temperature. After being washed in DPBS (-), samples were analyzed using an IN Cell Analyzer 6000 (GE Healthcare Life Sciences).

Sequencing

Genomic DNA was prepared from harvested cells using the DNeasy Blood & Tissue Kit (Qiagen, Cat. 69506) according to the manufacturer's instructions. sgRNA target sites were amplified by PCR using KAPA HiFi (KAPA Biosystems, Cat.KK2601). Primers are described in Table S4. PCR products were treated with EXOSAP-IT Express (Thermo Fisher Scientific, Cat.78201) prior to sequencing. DNA sequencing was performed using BigDye Terminator v3.1 Cycle Sequencing Kit (Applied Biosystems), purified by ethanol precipitation, and run on a 3130*xl* Genetic Analyzer (Applied Biosystems). Sequence alignments were performed using Snapgene v3.1.4. TIDE analysis of mixed sequences was performed using the online tool at https://tide.nki.nl/. The deletion size window was extended to 25 bp to accommodate larger deletions. The remaining parameters were set to default or allowed to adjust automatically based on the properties of the sequence trace files provided.

EdU staining and detection

EdU staining was conducted using Clik-iT® plus EdU flow cytometry assay kits (Thermo Fisher Scientific, Cat.C10424) according to the manufacturer's instructions. Briefly, cells were cultured with ES medium containing 10 µM EdU for 1 hour at d8 post-dox addition. Cells were harvested using TrypLE™ Select (1×) and fixed by paraformaldehyde contained Click-iT® fixative. Fixed cells were

permeabilized by Click-iT® saponin-based permeabilization and wash reagent, and incorporated EdU was stained with APC using Click-iT® reaction. The samples were analyzed using a BD LSRFortessa Cell Analyzer (BD Biosciences) with BD FACSDiva software (BD Biosciences). Flow cytometry data were analyzed and generated by FlowJo software.

Time-lapse video microscopy

For time-lapse imaging, wild type MEFs were transfected with PB-TAC-OK+9MS IRES mCherry nuclear membrane. The $TROP2^-$ and $TROP2^+$ reprogramming intermediates were isolated by cell sorting using BD FACSAria III cell sorter (BD Biosciences) on d6. 1×10^4 sorted cells were re-plated in ESC medium with 1 µg/mL of Doxycycline, anti SSEA-1 antibody conjugated with Brilliant Violet421 (BioLegend, Cat.125614, 1:200) and anti Trop2 antibody conjugated with FITC (R&D SYSTEMS, Cat.FAB1122F, 1:100) in µ-slide 8 well (ibidi, Cat.80826) with MEF feeder cells and cultured at 37°C. Each well was overlaid with silicon oil (Sigma-Aldrich, Cat.85419) and gassed on the microscope stage with 5% CO_2 and 5% O_2 using a self-developed continuous gas delivery lid system. Time-lapse imaging microscopy was carried out using Zeiss Axio Observer Z1 microscope (Zeiss) with definitive focus module, SpectraX light engine (Lumencor), 10x Fluar objective (Zeiss), transmitted VIS-LED (Zeiss) and an AxioCamHRm camera (1388x1040 pixel resolution). All hardware was controlled with a VBA module remote controlling Zeiss AxioVision 4.8.2 microscope control software (Filipczyk et al., 2015). Images were acquired on live cells at 50 ms in bright-field, 500 ms for the mCherry channel using an mCherry filter (Cat.F36-508, AHF), 500 ms for the SSEA1 Brilliant Violet421 channel using custom made DAPI filter (Cat, F39-404 for excitation and F47-460 for emission, AHF) and 500 ms for Trop2 FITC channel using EGFP filter (Cat.F46-002, AHF). Cells were imaged continuously at 30 min intervals for 3 days. The first image was acquired 2 hr after sorting. The medium was changed 21h and 43h after starting time-lapse movie. To edit time-lapse movies, tTt was used (Hilsenbeck et al., 2016).

Supplemental References

Filipczyk, A., Marr, C., Hastreiter, S., Feigelman, J., Schwarzfischer, M., Hoppe, P.S., Loeffler, D., Kokkaliaris, K.D., Endele, M., Schauberger, B.*, et al.* (2015). Network plasticity of pluripotency transcription factors in embryonic stem cells. Nat Cell Biol *17*, 1235-1246.

Hilsenbeck, O., Schwarzfischer, M., Skylaki, S., Schauberger, B., Hoppe, P.S., Loeffler, D., Kokkaliaris, K.D., Hastreiter, S., Skylaki, E., Filipczyk, A.*, et al.* (2016). Software tools for single-cell tracking and quantification of cellular and molecular properties. Nat Biotechnol *34*, 703-706.

Lichti, U., Anders, J., and Yuspa, S.H. (2008). Isolation and short-term culture of primary keratinocytes, hair follicle populations and dermal cells from newborn mice and keratinocytes from adult mice for in vitro analysis and for grafting to immunodeficient mice. Nat Protoc *3*, 799-810.

Ohnishi, K., Semi, K., Yamamoto, T., Shimizu, M., Tanaka, A., Mitsunaga, K., Okita, K., Osafune, K., Arioka, Y., Maeda, T.*, et al.* (2014). Premature termination of reprogramming in vivo leads to cancer development through altered epigenetic regulation. Cell *156*, 663-677.

Okita, K., Ichisaka, T., and Yamanaka, S. (2007). Generation of germline-competent induced pluripotent stem cells. Nature *448*, 313-317.

Ran, F.A., Hsu, P.D., Wright, J., Agarwala, V., Scott, D.A., and Zhang, F. (2013). Genome engineering using the CRISPR-Cas9 system. Nat Protoc *8*, 2281-2308.

Woltjen, K., Michael, I.P., Mohseni, P., Desai, R., Mileikovsky, M., Hamalainen, R., Cowling, R., Wang, W., Liu, P., Gertsenstein, M.*, et al.* (2009). piggyBac transposition reprograms fibroblasts to induced pluripotent stem cells. Nature *458*, 766-770.
