# Supplementary material for: OVOL1 Influences the Determination and Expansion of iPSC Reprogramming Intermediates
Source: Stem Cell Reports. 2019 Jan 10;12(2):319–32. doi: 10.1016/j.stemcr.2018.12.008 (PMC6372973; doi:10.1016/j.stemcr.2018.12.008)
Supplement: Document S2. Article plus Supplemental Information [file mmc7.pdf]

Stem Cell Reports
Article

OPEN ACCESS

OVOL1 Influences the Determination and Expansion of iPSC Reprogramming Intermediates

Harunobu Kagawa,[1] Ren Shimamoto,[2] Shin-Il Kim,[1] Fabian Oceguera-Yanez,[1] Takuya Yamamoto,[1] Timm Schroeder,[2] and Knut Woltjen[1,3,*]

[1]Department of Life Science Frontiers, Center for iPS Cell Research and Application (CiRA), Kyoto University, 53 Kawahara-cho, Shogoin, Sakyo-ku, Kyoto 606-8507, Japan
[2]Department of Biosystems Science and Engineering (D-BSSE), ETH Zurich, 4058 Basel, Switzerland
[3]Hakubi Center for Advanced Research, Kyoto University, Kyoto 606-8501, Japan
*Correspondence: woltjen@cira.kyoto-u.ac.jp
https://doi.org/10.1016/j.stemcr.2018.12.008

SUMMARY

During somatic cell reprogramming to induced pluripotent stem cells (iPSCs), fibroblasts undergo dynamic molecular changes, including a mesenchymal-to-epithelial transition (MET) and gain of pluripotency; processes that are influenced by Yamanaka factor stoichiometry. For example, in early reprogramming, high KLF4 levels are correlated with the induction of functionally undefined, transiently expressed MET genes. Here, we identified the cell-surface protein TROP2 as a marker for cells with transient MET induction in the high-KLF4 condition. We observed the emergence of cells expressing the pluripotency marker SSEA-1^{+} mainly from within the TROP2^{+} fraction. Using TROP2 as a marker in CRISPR/Cas9-mediated candidate screening of MET genes, we identified the transcription factor OVOL1 as a potential regulator of an alternative epithelial cell fate characterized by the expression of non-iPSC MET genes and low cell proliferation. Our study sheds light on how reprogramming factor stoichiometry alters the spectrum of intermediate cell fates, ultimately influencing reprogramming outcomes.

INTRODUCTION

Ectopic expression of OCT3/4, SOX2, KLF4m and c-MYC can convert somatic cells to induced pluripotent cells (iPSCs) (Takahashi and Yamanaka, 2006). At the molecular level, reprogramming processes starting from mouse embryonic fibroblasts (MEFs) can be divided into three phases: initiation, maturation, and stabilization (Samavarchi-Tehrani et al., 2010). The initiation phase is typically characterized by accelerated proliferation and the induction of a mesenchymal-to-epithelial transition (MET), while the maturation and stabilization phases are defined by entry into and acquisition of the pluripotency network (David and Polo, 2014). Since reprogramming is still a long and low-efficiency process, the high heterogeneity of reprogramming intermediates make it complicated to identify the true reprogramming paths and mechanisms (O'Malley et al., 2013; Polo et al., 2012). To overcome this, cell-surface antigens are relied upon as markers to predict bona fide reprogramming routes (Lujan et al., 2015; Zunder et al., 2015). However, cell-surface marker presentation is influenced by the induction method and resulting factor stoichiometry (Chantzoura et al., 2015). Even the surface presentation and dynamics of SSEA-1 (stage-specific embryonic antigen 1) (Andrews, 2011), an early pluripotency marker used for dissecting mouse reprogramming, is diverse among different reprogramming systems (Kim et al., 2015; Polo et al., 2012).

The development of polycistronic systems allowed researchers to induce a prescribed stoichiometry of Yamanaka factors among transduced cells, with the intention of reducing heterogeneity of reprogramming intermediates and, in turn, the complexity of reprogramming (Carey et al., 2009; Sommer et al., 2009). Among the Yamanaka factors, KLF4 plays important roles for both MET induction and the acquisition of pluripotency (Carey et al., 2011; Li et al., 2010; Polo et al., 2012; Sridharan et al., 2009). We previously identified that an N-terminal 9-amino-acid difference in *Klf4* cDNAs commonly employed in polycistronic cassettes affects the final stoichiometry of reprogramming factors (Kim et al., 2015). In general, polycistronic cassettes utilizing short *Klf4* (OKMS, STEMCCA, WTSI, and EB-C5) (Chou et al., 2011; Kim et al., 2015; Sommer et al., 2009; Yusa et al., 2009) induce low KLF4 protein expression compared with cassettes that utilize long *Klf4* (OK^{+9}MS, OSKM, and MKOS) (Carey et al., 2009; Kaji et al., 2009; Kim et al., 2015) and induce high KLF4 protein expression. This difference in KLF4 consistently results in the induction of dissimilar reprogramming paths and efficiencies (Kim et al., 2015).

Critically, high-KLF4 achieves efficient reprogramming compared with low-KLF4 (Kim et al., 2015). During high-KLF4 reprogramming we observed the expression of MET genes sustained in the pluripotent state, such as *Epcam* and *Cdh1*, in addition to transiently upregulated epithelial genes (Kim et al., 2015). Transient upregulation of lineage-specific genes has been observed using different reprogramming systems (Cacchiarelli et al., 2015; Nefzger et al., 2017; Polo et al., 2012; Takahashi et al., 2014). However, the role of transient gene activation in determining cellular and molecular reprogramming phenotypes, and

This is an open access article under the CC BY license (http://creativecommons.org/licenses/by/4.0/).

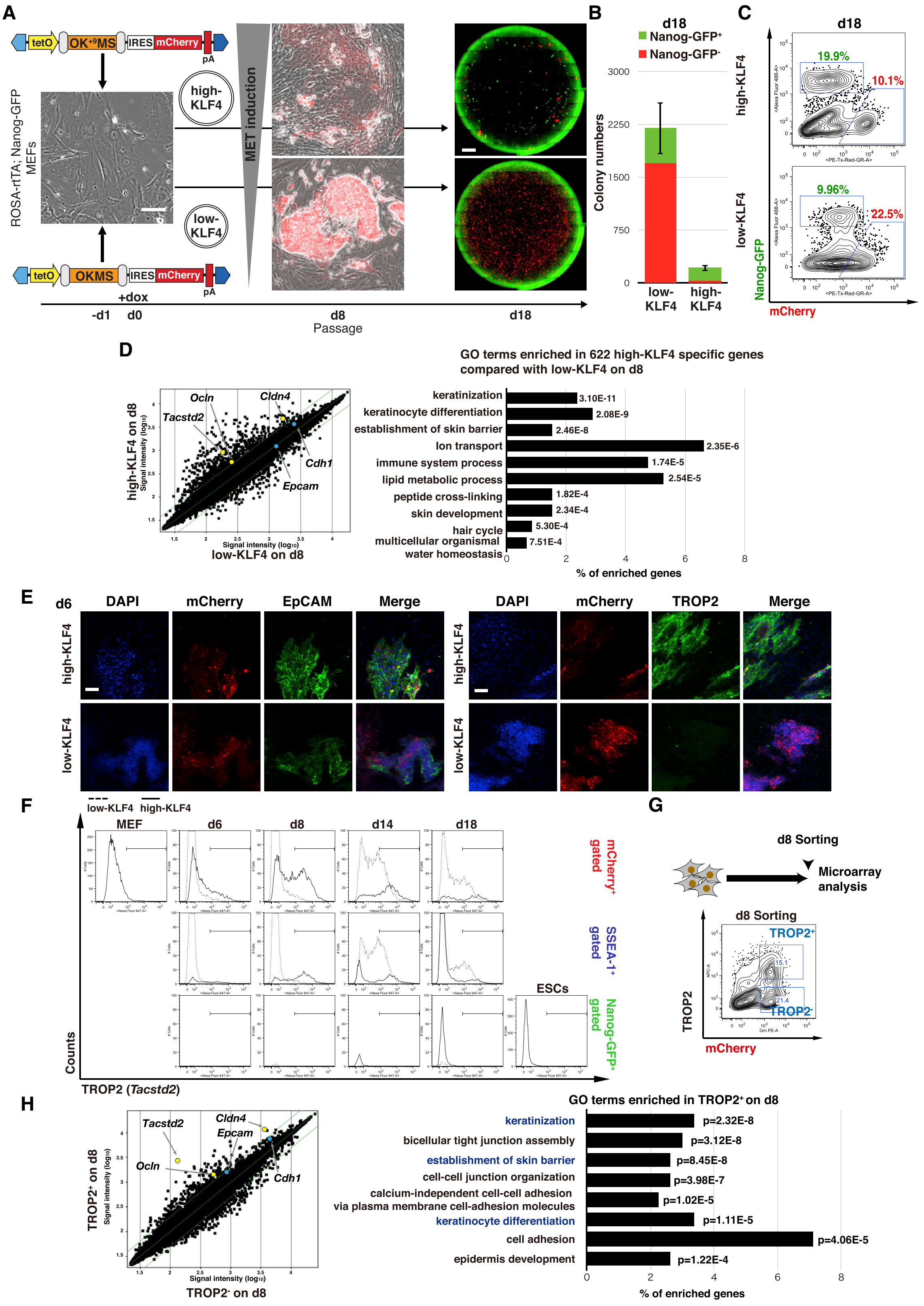

(legend on next page)

ultimately the acquisition of pluripotency, is still not fully appreciated.

In this study, we distinguish between the sustained and transient MET genes based on their expression dynamics, and aim to ascribe a functional role of transient MET induction in defining high-KLF4 reprogramming characteristics. We identify TROP2 as a cell-surface marker for transient MET and reprogramming potential. Using a focused CRISPR screen tracking TROP2 expression, we reveal OVOL1 as a candidate regulator of transient MET induction. Further functional analyses suggest that OVOL1 is not directly implicated in the acquisition of pluripotency, but rather acts to repress proliferation and the expansion of cells that fail to reprogram, helping to explain the apparent high efficiency of reprogramming with high-KLF4 stoichiometry.

RESULTS

Identification of a Cell-Surface Marker for KLF4-Induced Epithelialization

MET genes activated early in reprogramming including *Epcam* and *Cdh1*, which sustain their expression in iPSCs, promoting the reprogramming process (Kuan et al., 2017; Li et al., 2010). Based on transient MET gene expression and consistent pluripotency activation observed respectively in early and late high-KLF4 reprogramming, we hypothesized that aspects of transient MET induction may exert a positive effect on reprogramming. To test our hypothesis, we first compared low-KLF4 (OKMS) and high-KLF4 ($OK^{+9}MS$) reprogramming conditions (Figure 1A). Reprogramming was initiated by doxycycline treatment on day 0 and cells were harvested for flow-cytometry analysis on day 8, then passaged for analysis on day 18. Intermediate cells were tracked by transgene-linked expression of mCherry, while full reprogramming was detected by activation of the pluripotency reporter Nanog-GFP and concurrent silencing of mCherry (Figure 1A). As noted previously (Kim et al., 2015), expansion of $mCherry^+$ cells and acquisition of SSEA-1 were both reduced in the high-KLF4 condition compared with low-KLF4 (Figure S1A). Yet, the majority of colonies on day 18 successfully silenced exogenous mCherry and acquired Nanog-GFP reporter expression (Figures 1A–1C), indicating the efficient reprogramming of high-KLF4. Despite robust reprogramming initiation, most low-KLF4 colonies did not silence mCherry and remained $Nanog\text{-}GFP^-$ on day 18, resulting in low-efficiency reprogramming due to an expansion of $mCherry^+$ cells (Figures 1B and 1C).

At the molecular level, high-KLF4 induces epithelial and epidermal genes that are not expressed by MEFs or the resulting iPSCs (Kim et al., 2015). The 622 genes upregulated more than 2-fold on day 8 in high-KLF4 compared with

Figure 1. Classification of Genes Upregulated in the High-KLF4 Intermediates

(A) Scheme depicting reprogramming with OKMS (low-KLF4) or $OK^{+9}MS$ (high-KLF4) polycistronic cassettes and analysis of their characteristics. Polycistronic cassettes were delivered by a *piggyBac* (PB) transposon with mCherry into ROSA-rtTA Nanog-GFP MEFs (-d1). Cultures were passaged on day 8 and the reprogramming capacity was analyzed on day 18. See main text for further details. Blue polygons represent PB 3′ (left) and 5′ (right) inverted terminal repeats. tetO, doxycycline-responsive promoter; IRES, internal ribosome entry signal; pA, polyadenylation signal. Microscopy image (left) shows the representative morphology of MEFs and intermediate colonies. Scale bars, 100 μm. Whole-well fluorescence microscopy images (right) on day 18 for Nanog-GFP and mCherry from low- and high-KLF4. Scale bars, 4,000 μm.

(B) Quantification of $Nanog\text{-}GFP^-$ and $Nanog\text{-}GFP^+$ colony numbers on day 18 in low- and high-KLF4. Means ± SD for total colonies from three independent experiments.

(C) Flow-cytometry analysis on day 18 for Nanog-GFP and mCherry in low- and high-KLF4.

(D) (Left) Correlation plot for gene expression in $mCherry^+$ sorted populations from low- and high-KLF4 on day 8. Green lines indicate 2-fold changes. Genes related to sustained and transient MET genes are highlighted (yellow, >2-fold; blue, <2-fold) Signal intensity values are average of two independent experiments. (Right) Gene ontology (GO) term analysis for genes expressed 2-fold higher in the high-KLF4 reprogramming, arranged in order of p value and indicating the proportion of genes represented for each enriched GO term. Cutoff $p = 1.0 \times 10^{-3}$.

(E) Immunofluorescence antibody staining for EpCAM and TROP2 in low- and high-KLF4 on day 6. Green staining shows EpCAM (left) and TROP2 (right), respectively. DAPI staining indicates nuclear density. Reprogramming cells are visualized by mCherry fluorescence. Scale bar, 100 μm.

(F) Flow-cytometry analysis of TROP2 expression dynamics. Histograms are grouped by analysis day (columns) and population gating (rows). Dashed lines and straight lines represent low-KLF4 and high-KLF4, respectively.

(G) Gating scheme for TROP2 cell sorting from high-KLF4 reprogramming on day 8.

(H) (Left) Correlation plot for gene expression in day 8 $TROP2^+$ and $TROP2^-$ sorted populations. Green lines indicate 2-fold changes. Genes related to sustained and transient MET genes are highlighted (yellow, >2-fold; blue, <2-fold). (Right) GO term analysis for genes expressed 2-fold higher in the $TROP2^+$ population, arranged in order of p value and indicating the proportion of genes represented for each enriched GO term. GO terms common with (D) are highlighted in blue.

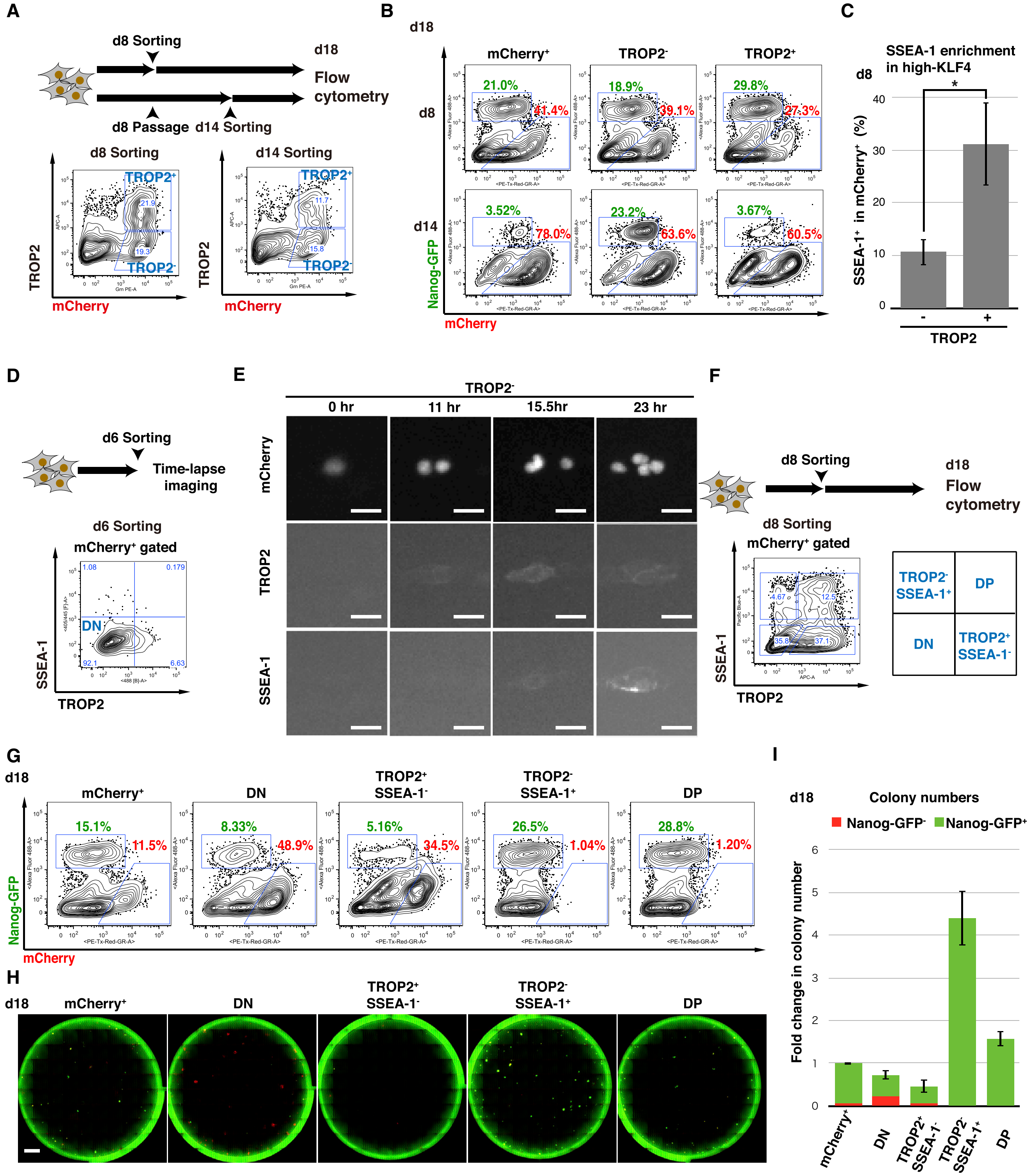

Figure 2. Transient TROP2 Is an Earlier Marker than SSEA-1

(A) Gating scheme for TROP2 cell sorting from high-KLF4 reprogramming on day 8 and day 14.

(B) Flow-cytometry analysis on day 18 for Nanog-GFP and mCherry from each day 8 or day 14 sorted population.

(C) Proportion of SSEA-1^+ cells in $TROP2^-$ and $TROP2^+$ populations at day 8 of high-KLF4 reprogramming. Means ± SD for eight independent experiments. *$p < 0.05$, Student's t test.

(legend continued on next page)

low-KLF4 included *Ocln* and *Cldn4* and were enriched in keratinocyte and skin development gene ontology (GO) terms (Figure 1D). Analysis of microarray data on days 2, 4, 6, 8, and 18 compared with MEF, iPSCs, and mouse embryonic stem cells (mESCs) revealed that these genes were transiently upregulated in the early phase of high-KLF4 reprogramming (Figure S1B). Of note, expression of sustained MET genes *Epcam* and *Cdh1* were similar between the low- and high-KLF4 conditions (Figure 1D). Taken together, transient MET genes were specifically upregulated during the early phase of high-KLF4 reprogramming.

The heterogeneity of reprogramming intermediates can be resolved using appropriate cell-surface markers (Buganim et al., 2012; Polo et al., 2012). We therefore aimed to identify cell-surface markers associated with transient MET. From the 622 high-KLF4 specific genes (Figure 1D and Table S1), we assessed membrane proteins including integrin subunit β4 (ITGB4) and 5′-nucleotidase ecto (NT5E), which were used previously to plot reprogramming trajectories by mass cytometry (Lujan et al., 2015; Zunder et al., 2015), integrin subunit α6 (ITGA6), which is known to heterodimerize with β subunit ITGB4 or ITGB1 as a receptor for laminin (Takada et al., 2007), and TROP2, encoded by tumor-associated calcium signal transducer 2 (*Tacstd2*), with high amino acid sequence similarity (67%) to EpCAM (McDougall et al., 2015). Consistent with the microarray data (Figure 1D), EpCAM protein expression was detected by immunofluorescence in both low- and high-KLF4 on day 6, whereas TROP2 was only detected in high-KLF4 (Figure 1E). In the high-KLF4 condition, flow cytometry revealed that all markers were acutely upregulated in the early phase of reprogramming, while only EpCAM and TROP2 presented a bimodal distribution pattern (Figures 1F and S1C–S1F). Curiously, in contrast to the intermediate expression of EpCAM in low-KLF4 on day 8 (median fluorescence intensity [MFI], 3,500), expression in high-KLF4 reprogramming was bimodal, yet EpCAM-positive cells were more intense (MFI, 8,481) than that of mESCs (MFI 3,259). In cells from high-KLF4 which went on to acquire Nanog-GFP, EpCAM expression was reduced to mESC levels (MFI, 3,788) (Figure S1C), implying a fundamental difference in MET induction between low- and high-KLF4. TROP2 was specifically upregulated in high-KLF4 compared with low-KLF4 in the early phase (days 6–8), overlapping in part with SSEA-1 expression, but not with Nanog-GFP (Figure 1F). Only late mCherry^{+} cells retained TROP2 marker expression on day 18.

Supporting the connection between KLF4 stoichiometry and transient MET with TROP2 presentation, we observed a similar pattern of high TROP2 and low SSEA-1 among other high-KLF4 reprogramming systems (OSKM and MKOS) while low-KLF4 systems (STEMCCA, WTSI and EB-C5) had low TROP2 and high SSEA-1 (Figure S1G). To further characterize TROP2^{-} and TROP2^{+} populations, we carried out cell sorting at day 8, followed by global gene expression analysis (Figures 1G and S1H). Overall, the list of >2-fold upregulated genes in the TROP2^{+} population significantly enriched the GO terms related to biological processes of epithelial development and cell adhesion including “keratinization,” “establishment of skin barrier,” and “keratinocyte differentiation,” reminiscent of GO terms enriched in the high-KLF4 specific gene list and indicating enrichment from the total mCherry^{+} population (Figures 1D and 1H; Table S1). Moreover, *Epcam* and *Cdh1* displayed higher expression in TROP2^{+} than TROP2^{-}, although the difference was only slightly more than 1.5-fold (Figure 1H). Based on these results, we propose TROP2 as a candidate marker for identification of the transient MET population in the early phase of high-KLF4 reprogramming.

Evaluation of TROP2 as a Marker for Reprogramming

Next, we compared the reprogramming capacity of TROP2^{+} and TROP2^{-} populations from early (day 8) and late (day 14) reprogramming by cell sorting and extended culture (Figure 2A). By day 18, the Nanog-GFP^{+} proportion in the day 8-sorted TROP2^{+} culture was 1.5-fold higher than that of TROP2^{-} (29.8% versus 18.9%). On the contrary, TROP2^{+} sorting on day 14 could not enrich for cells with a high reprogramming capacity, while TROP2^{-} sorting showed a positive enrichment (3.67% versus 23.3%) (Figure 2B). Interestingly, flow-cytometry analysis of high-KLF4 at day 8 revealed that SSEA-1^{+} cells are enriched nearly 3-fold in the TROP2^{+} population compared with TROP2^{-} (Figure 2C). These results suggest that TROP2 expression in the early phase indicates reprogramming

(D) Gating scheme for TROP2^{-}SSEA-1^{-} (DN) cell sorting from high-KLF4 reprogramming on day 6.

(E) Live fluorescence microscopy images of nuclear mCherry, TROP2, and SSEA-1 at the indicated time points after cell sorting and replating of TROP2^{-} cells on day 6. Scale bars, 25 μm.

(F) Gating scheme for DN, TROP2^{+}SSEA-1^{-}, TROP2^{-}SSEA-1^{+}, and DP cell sorting from high-KLF4 reprogramming on day 8.

(G) Flow-cytometry analysis on day 18 for Nanog-GFP and mCherry from each day 8 sorted population.

(H) Whole-well fluorescence microscopy images on day 18 for Nanog-GFP and mCherry from each day 8 sorted population. Scale bars, 4,000 μm.

(I) Quantification of Nanog-GFP^{-} and Nanog-GFP^{+} colony numbers at day 18 from each day 8 sorted population. All colony numbers are normalized to mCherry^{+}. Means ± SD for three independent experiments.

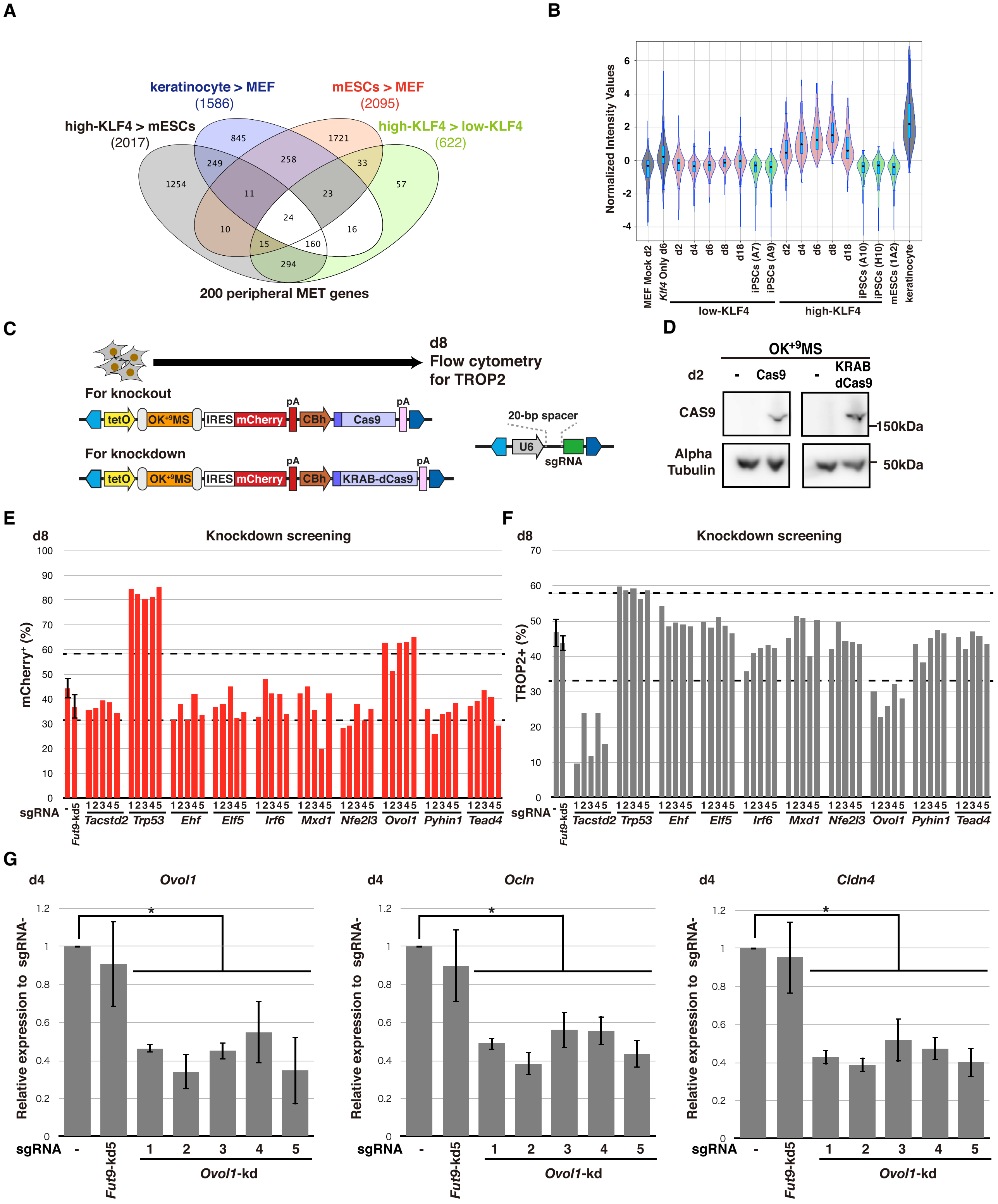

Figure 3. CRISPR/Cas Screening Reveals OVOL1 as a Transient MET Regulator at Reprogramming Initiation

(A) Venn diagram from day 8 microarray analysis. Total numbers of genes with a 2-fold expression difference in crosswise comparisons of mESCs versus MEFs (red), high-KLF4 versus low-KLF4 (green), keratinocytes versus MEFs (blue), or high-KLF4 versus mESCs (gray) are shown.

(legend continued on next page)

progression, whereas cells that retain TROP2 expression in the late phase lose their reprogramming capacity.

In contrast to EpCAM, which is expressed earlier and more uniformly in SSEA-1-positive cells from high-KLF4 (Figure S1C), the bimodal distribution pattern of TROP2 in both mCherry$^+$ and SSEA-1$^+$ cell fractions (Figure 1F) as well as a similar timing of emergence on day 8 prevented us from determining the timing of cell-surface marker transition. To clarify the order of TROP2 and SSEA-1 presentation, we isolated double-negative (DN) cells on day 6 and performed live antibody staining with time-lapse microscopy over 3 days (Figure 2D and Video S1). Eleven hours after sorting, mCherry$^+$ cells started to express TROP2 but remained SSEA-1 (Figure 2E). An additional ~6–12 hr later, TROP2$^+$ colonies (18/20) started to express SSEA-1, with an apparent decrease in TROP2. Similarly, sequential cell-surface marker presentation was observed for TROP2$^+$ cells (Video S1). These results indicate that the majority of high-KLF4 reprogramming cells sequentially present TROP2 and then SSEA-1.

To functionally evaluate the conversion from TROP2$^+$ toward SSEA-1$^+$ as predictors of reprogramming capacity in the high-KLF4 condition, we performed cell sorting on day 8 for DN, TROP2$^+$SSEA-1$^-$, TROP2$^-$SSEA-1$^+$, and double-positive (DP) populations (Figures 2F and S2A). We then quantified the reprogramming capacity of sorted cells by continuous culture in parallel with bulk mCherry$^+$ cells. Proportions of Nanog-GFP$^+$ and mCherry$^+$ cells on day 18 revealed that DN and TROP2$^+$SSEA-1$^-$ populations possessed a lower capacity to become Nanog-GFP$^+$ than the bulk mCherry$^+$ population (Figure 2G), indicating that a majority of cells from these populations fail to reprogram, a conclusion supported by the retention of mCherry$^+$ expression in cultures from DN and TROP2$^+$SSEA-1$^-$ fractions (Figure 2G). Although the proportion of mCherry$^+$ cells was comparable between DN and TROP2$^+$SSEA-1$^-$ populations, total colony numbers and cell numbers were limited in TROP2$^+$SSEA-1$^-$ population (Figures 2H, 2I, and S2B). On the contrary, TROP2$^-$SSEA-1$^+$ and DP populations possessed a higher capacity to produce Nanog-GFP$^+$ cells (26.5% and 28.8%, respectively) with essentially no mCherry$^+$ cells (<1.5%) (Figure 2G), indicating that SSEA-1 is a predictive marker of reprogramming in the high-KLF4 condition. Consistent with the difference between DN and TROP2$^+$SSEA-1$^-$, total Nanog-GFP$^+$ colony numbers for TROP2$^-$SSEA-1$^+$ were nearly double that of DP (Figures 2H and 2I). These results support that cell-surface marker transition (TROP2$^+$SSEA-1$^-$ > DP > TROP2$^-$SSEA-1$^+$) is required for successful reprogramming progression, as predicted by live cell imaging (Figure 2E). Moreover, these data indicate that TROP2 may be used to identify a distinct intermediate cell population in reprogramming, prior to SSEA-1 presentation.

Identification of Transient MET Regulators by CRISPR/Cas9 Candidate Screen

To evaluate the role of the transient MET response in the acquisition of pluripotency, we aimed to identify transcriptional regulators of high-KLF4 MET induction among transiently upregulated genes (Figure 1D). We further refined the list of 622 high-KLF4 specific genes (Figure 3A, green) through comparison with keratinocyte (blue, included) or mESC (red, excluded) gene lists. As a result, 176 genes were identified (160 + 16). An additional 24 genes shared between high-KLF4, keratinocytes, and mESCs were also included, as their expression in high-KLF4 reprogramming on day 8 transiently exceeded that of mESCs (Figure 3A), resulting in a total of 200 genes (Table S2). Furthermore, transient expression specific to the high-KLF4 condition was confirmed throughout the 18-day reprogramming period (Figure 3B), where expression peaked at day 8 and was retained in mCherry$^+$ cells on day 18, while iPSCs silenced gene expression similarly to mESCs. Eight genes encoding transcription factors (*Ehf*, *Elf5*, *Irf6*, *Mxd1*, *Nfe2l3*, *Ovol1*, *Pyhin1*, and *Tead4*) were selected from the list of 200 transient MET genes using the GO term “sequence-specific

(B) Violin plot of normalized intensity values for transient MET genes in low- or high-KLF4 reprogramming processes. The box plot displays the median, 25th, and 75th percentiles.

(C) Addition of constitutive CBh promoter-driven Cas9 or KRAB-dCas9 with FLAG tag on N-terminal region to PB reprogramming vectors. PB vectors for U6 promoter-driven sgRNA expression were constructed and delivered separately.

(D) Western blot analysis of CAS9 and KRAB-dCAS9 in OK^{+9}MS (high-KLF4)-transfected MEFs cultured for 2 days with or without CBh-driven Cas9 or KRAB-dCas9. A-Tubulin was used as a loading control. Data for additional days are provided in Figure S3A.

(E) Proportions of mCherry$^+$ cells by flow-cytometry analysis on day 8 with target gene knockdown. Dotted lines show the mean for sgRNA$^-$ ± 3 SD used as screening thresholds. Means ±SD for ten independent experiments for sgRNA$^-$ and *Fut9* sgRNA samples. For other sgRNAs, n = 1.

(F) Proportions of TROP2$^+$ cells by flow-cytometry analysis on day 8 with target gene knockdown. Dotted lines show the mean for sgRNA$^-$ ± 3 SD used as screening thresholds. Means ± SD for ten independent experiments for sgRNA$^-$ and *Fut9* sgRNA samples. For other sgRNAs, n = 1.

(G) qRT-PCR analysis for *Ovol1*, *Cldn4*, and *Ocln* following *Ovol1* knockdown in high-KLF4 on day 4. *Fut9*-kd5 was used as a control. All mRNA levels are normalized to Gapdh and relative to sgRNA$^-$. Means ± SD for three independent experiments. *p < 0.05. Student’s t test.

DNA binding transcription factor activity." Interestingly, most of the candidate transcription factors, including EHF (Albino et al., 2012), ELF5 (Chakrabarti et al., 2012), IRF6 (Richardson et al., 2006), MXD1 (Werner et al., 2001), NFE2L3 (Chevillard and Blank, 2011), OVOL1 (Roca et al., 2013), and TEAD4 (Zhang et al., 2011), are expressed in the epidermis with known roles in maintaining epithelial cell characteristics or regulating wound healing. Our analysis, therefore, established a list of genes expressed in the high-KLF4 condition, which are unique and transient compared with sustained MET genes.

We employed *Streptococcus pyogenes* CRISPR/Cas (Cas9) nuclease and interference systems for selected transcription factor gene knockout or knockdown during reprogramming (Figure 3C). To ensure that Cas9 activity was limited to reprogramming cells, we included a CBh promoter-driven expression cassette for Cas9 nuclease or the KRAB repression domain fused to nuclease-dead Cas9 (KRAB-dCas9) within the PB reprogramming vectors (Figure 3C). CAS9 and KRAB-dCAS9 protein expression in both low-KLF4 and high-KLF4 conditions were detected by western blot in bulk culture on day 2 (Figure 3D). Protein levels for CAS9 and KRAB-dCAS9 decreased after day 4 in the high-KLF4 condition and after day 8 for low-KLF4 (Figure S3A). As a demonstration of Cas9- and KRAB-dCas9-mediated gene regulation, we targeted α1,3-fucosyltransferase IX (*Fut9*), the key enzyme for SSEA-1 synthesis (Kudo et al., 2004). Previously published single guide RNA (sgRNA) libraries were used to select *Fut9* sgRNA sequences (*Fut9*-ko1–ko5 for knockout and *Fut9*-kd1, -kd2, -kd4, and -kd5 for knockdown, Figure S3B) (Horlbeck et al., 2016; Koike-Yusa et al., 2014). Both systems robustly suppressed SSEA-1 presentation on day 8 (Figure S3C). Comparison of day 8 with day 18 revealed that, in marked contrast to the permanent loss of SSEA-1 by Cas9-mediated gene knockout, KRAB-dCas9-mediated gene knockdown diminished over time due to the silencing of KRAB-dCas9 expression in the later phase of reprogramming (Figure S3D). Finally, we confirmed that expression of Cas9 or KRAB-dCas9, with or without *Fut9* sgRNAs, has no effect on reprogramming efficiency by assessing mCherry and Nanog-GFP reprogramming outcomes (Figure S3E).

Acute early-phase KRAB-dCas9 knockdown was deemed appropriate for screening transient MET regulators, while avoiding potentially complex phenotypes arising from a diverse mutation spectrum induced by Cas9 (Mandegar et al., 2016). We employed TROP2 as a measure of perturbed transient MET induction, and screened five different sgRNAs (kd1–kd5) per candidate gene at the start of high-KLF4 KRAB-dCas9 reprogramming, with no-sgRNA (sgRNA$^-$) or *Fut9*-kd5 transfected cells as negative controls. We included sgRNAs for *Trp53* and *Tacstd2* in the screen as positive controls, as p53 suppresses cell proliferation mediated by p21 during reprogramming (Hong et al., 2009). At day 8, we assessed differences in mCherry$^+$ and TROP2$^+$ proportions by flow-cytometry analysis. All five *Trp53* sgRNAs induced a 35%–40% increase in mCherry$^+$ cells at day 8 (Figure 3E), suggesting increased cell proliferation consistent with previous reports. Furthermore, all five *Tacstd2* sgRNAs decreased the TROP2$^+$ cell population by 20%–35% without affecting the mCherry$^+$ proportion (Figures 3E and 3F), suggesting that *Tacstd2* itself may not have any functional role in early reprogramming. Among the eight selected genes, only *Ovol1* knockdown consistently resulted in a statistically significant increase in the mCherry$^+$ population (Figures 3E, S4A, and S4B) and reduction in the TROP2$^+$ population (Figures 3F and S4C).

Given the results from TROP2-based screening and known role of OVOL1 in regulating epidermal differentiation (Lee et al., 2014), we asked whether knockdown of *Ovol1* also inhibits the induction of transient MET genes. Using qRT-PCR we analyzed the expression of *Cldn4* and *Ocln*, representative transient MET genes in high-KLF4 (Table S2) previously described in the initial characterization of the MET process (Li et al., 2010; Samavarchi-Tehrani et al., 2010). qRT-PCR analysis revealed that *Ovol1* suppression by knockdown was accompanied by a significant decrease in both *Cldn4* and *Ocln* expression (Figure 3G). Interestingly, in response to *Ovol1* knockdown, EpCAM expression levels measured by flow-cytometry analysis decreased to match the expression levels observed in mESCs (Figure S4D). Overall, these results suggest that OVOL1 regulates aspects of transient MET that contribute to the identity of high-KLF4 reprogramming intermediates.

Ovol1 Knockdown Increases Both Failed and Fully Reprogrammed Colony Numbers

Next, we aimed to reveal the effect of *Ovol1* knockdown on final reprogramming efficiencies. In contrast to our initial hypothesis that transient MET induction may have a positive effect on the acquisition of pluripotency, no obvious change was observed in the proportion of Nanog-GFP$^+$ cells by flow-cytometry analysis, while mCherry$^+$ cells increased substantially (Figures 4A and S5A). Interestingly, quantification of whole-well images from day 18 cultures revealed a significant increase in Nanog-GFP$^+$ and large mCherry$^+$ colonies, a result consistent across all five *Ovol1* sgRNAs (Figures 4B, 4C, and S5B) and corroborating the increase in mCherry$^+$ cells noted at day 8 in the knockdown screen.

To validate our knockdown data, we performed *Ovol1* knockout using Cas9. We selected three sgRNAs (*Ovol*-ko1, -ko2, and -ko5) based on their target sites (Figure S4A). Consistent with the knockdown result, *Ovol1* knockout with any of the three sgRNAs led to an overall increase in both mCherry$^+$ and Nanog-GFP$^+$ colonies on day 18, while by flow-cytometry analysis the proportion of mCherry$^+$

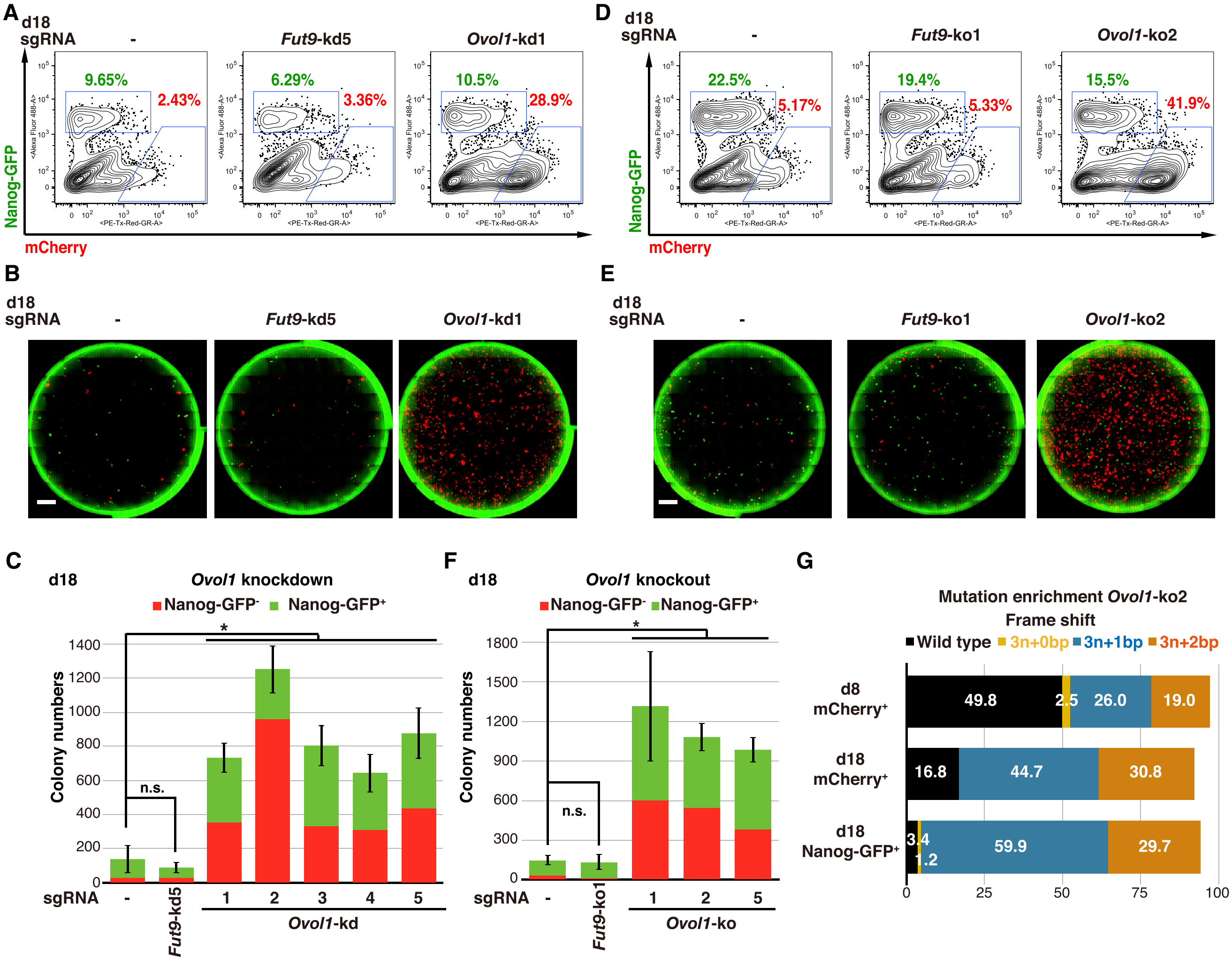

Figure 4. *Ovol1* Knockdown Increases the Total Colony Numbers

(A) Flow-cytometry analysis on day 18 for Nanog-GFP and mCherry following *Ovol1* knockdown in high-KLF4. See also Figure S5A.

(B) Whole-well fluorescence microscopy images for Nanog-GFP and mCherry on day 18 following *Ovol1* knockdown in high-KLF4. Scale bars, 4,000 μm. See also Figure S5B.

(C) Quantification of Nanog-GFP^{-} and Nanog-GFP^{+} colony numbers on day 18 following *Ovol1* knockdown in high-KLF4. Means ± SD for total colonies from four independent experiments. *$p < 0.05$. Student's t test.

(D) Flow-cytometry analysis on day 18 for Nanog-GFP and mCherry following *Ovol1* knockout in high-KLF4. See also Figure S5C.

(E) Whole-well fluorescence microscopy images for Nanog-GFP and mCherry on day 18 following *Ovol1* knockout in high-KLF4. Scale bars, 4,000 μm. See also Figure S5D.

(F) Quantification of Nanog-GFP^{-} and Nanog-GFP^{+} colony numbers on day 18 following *Ovol1* knockout in high-KLF4. Means ± SD for total colonies from three independent experiments. *$p < 0.05$. Student's t test.

(G) Distribution of mutation frequencies predicted by TIDE in day 8 mCherry^{+}, day 18 mCherry^{+}, and day 18 Nanog-GFP^{+} populations following *Ovol1* knockout in high-KLF4. Mutant alleles were categorized based on a resulting in-frame indels 3n + 0 bp (yellow), frameshifted indels 3n + 1 bp (blue), or 3n + 2 bp (orange). Wild-type (black) alleles showed no indel. Data are representative of two independent experiments.

See also Figure S5E.

cells grew measurably (Figures 4D–4F, S5C, and S5D). To reveal the link between insertion or deletion mutations (indels) and phenotype, we performed cell sorting for mCherry^{+} on day 8 and for mCherry^{+} or Nanog-GFP^{+} on day 18, followed by PCR and Sanger sequencing of target sites. We estimated indel types and frequencies in each population by employing computational sequence trace decomposition (TIDE) from mixed PCR amplicons

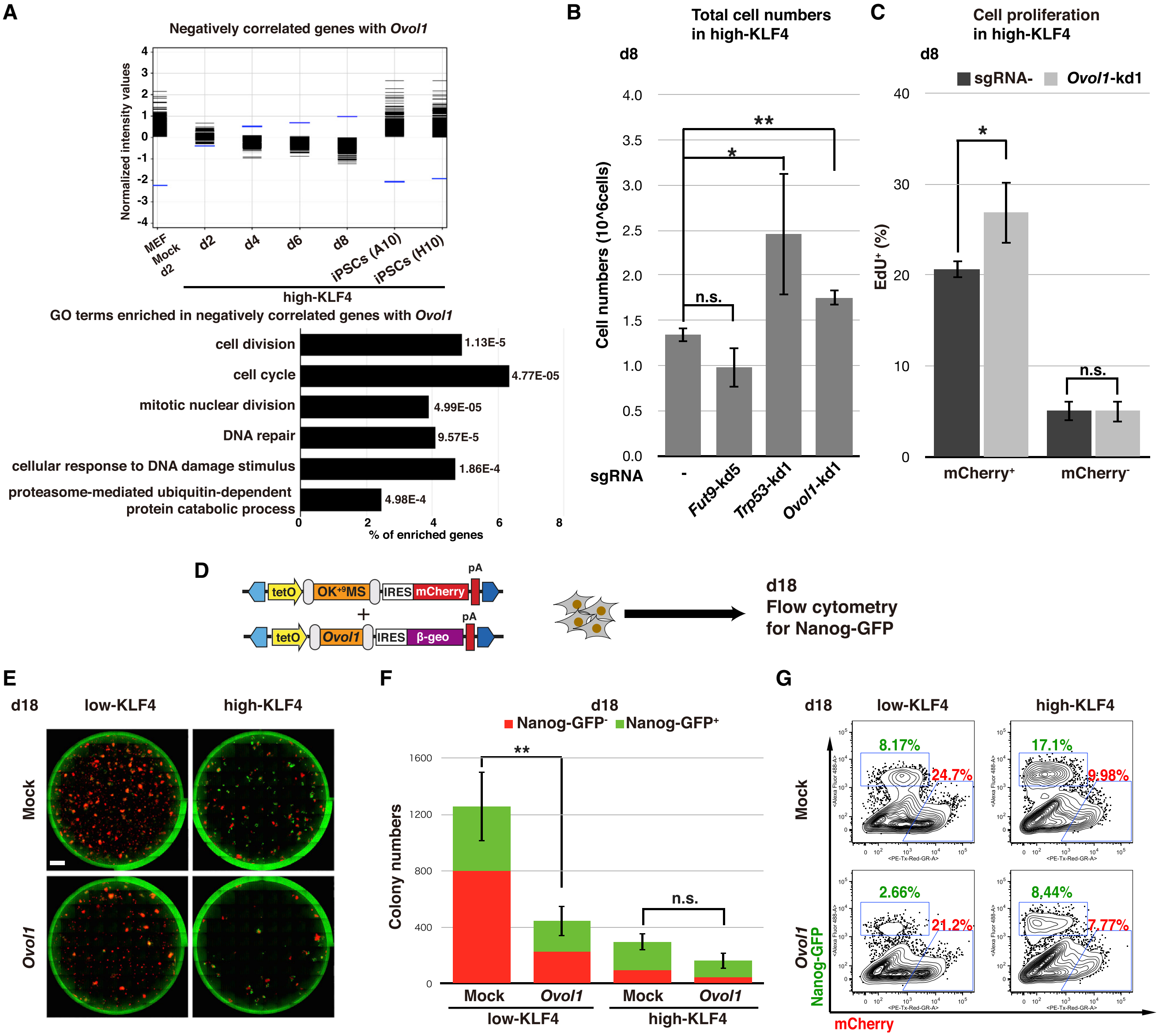

Figure 5. OVOL1 Regulates Cell Proliferation in High-KLF4 Intermediates

(A) (Top) Expression level of 607 genes (black), which are negatively correlated with *Ovol1* expression (blue) during high-KLF4 reprogramming. The gene set was derived using GeneSpring's "find similar entities" analysis with a correlation cutoff range of $-1.0 \leq r \leq -0.9$. (Bottom) GO term analysis of negatively correlated genes, arranged in order of p value and indicating the proportion of genes represented for each enriched GO term.

(B) Total cell numbers on day 8 following gene knockdown in high-KLF4. Means ± SD for three independent experiments (Student's t test for each sgRNA versus sgRNA^{-}, *Fut9*-kd5, $p = 0.056$; *Trp53*-kd1, $p = 0.047$; *Ovol1*-kd1, $p = 0.0043$). $*p < 0.05$, $**p < 0.01$.

(C) Flow-cytometry analysis of EdU incorporation in mCherry^{+} and mCherry^{-} cells for each sgRNA^{-} (black) and *Ovol1*-kd1 (gray) in high-KLF4 on day 8. Means ± SD for three independent experiments (Student's t test *Ovol1*-kd1 versus sgRNA^{-} in each mCherry^{+} and mCherry^{-}, in mCherry^{+}, $p = 0.038$; in mCherry^{-}, $p = 0.93$). $*p < 0.05$.

(D) Scheme for *Ovol1* overexpression experiments. PB-TAB expression vectors were co-transfected with OKMS (low-KLF4) or OK^{+9}MS (high-KLF4) plasmid. Reprogramming efficiencies were analyzed on day 18 without day 8 passage.

(E) Whole-well fluorescence microscopy images for Nanog-GFP and mCherry on day 18 following overexpression of *Ovol1* in low- and high-KLF4. Scale bars, 4,000 μm.

(legend continued on next page)

(Brinkman et al., 2014), and classified the results based on no mutation (WT), in-frame indels (3n + 0 bp), or frameshifted indels (3n + 1 or +2 bp). Indel rates in the mCherry$^+$ fraction ranged from 50% (*Ovol*-ko2) to 90% (*Ovol*-ko5) on day 8 (Figures 4G and S5E). Intriguingly, indels resulting in a 1- or 2-bp frameshift were enriched in both the mCherry$^+$ and Nanog-GFP$^+$ populations by day 18, while WT alleles were depleted in both populations across all three sgRNA treatments (Figures 4G and S5E), indicating that not only is OVOL1 activity unnecessary for the acquisition of pluripotency, but its knockout also provides a selective advantage to reprogramming intermediates. For *Ovol1*-ko1, zero-frameshift indels were also observed to increase in both day 18 populations (Figure S5E), highlighting the importance of the OVOL1 zinc-finger domains in mediating the OVOL1 phenotype (Figure S4A). Taken together, these results indicate that OVOL1 suppression allows for an overall higher proliferation rate in high-KLF4 reprogramming, with a particularly strong effect on the expansion of failed reprogramming cells.

OVOL1 Regulates Proliferation of Intermediates in High-KLF4 Reprogramming

Finally, we aimed to identify the mechanism by which *Ovol1* knockdown results in a robust expansion of mCherry$^+$ cells. OVOL1 is known to suppress target genes through histone deacetylase recruitment (Nair et al., 2007). Based on this repressor function of OVOL1, we identified genes that were negatively correlated with *Ovol1* expression in the high-KLF4 condition. Negatively correlated genes showed an enrichment of GO terms related to cell proliferation including “cell division,” “cell cycle,” and “mitotic nuclear division” (Figure 5A and Table S3). These results are consistent with binding of OVOL1 to the *c-Myc* promoter in overexpression assays (Nair et al., 2006), and an observed increase in mCherry$^+$ cells at day 8 and total colony numbers at day 18 following *Ovol1* knockdown (Figures 3E and 4C). Moreover, total cell numbers on day 8 following *Trp53* or *Ovol1* knockdown were significantly increased compared with sgRNA$^-$ or *Fut9* knockdown (Figure 5B). 5-Ethynyl-2′-deoxyuridine (EdU) labeling for proliferating cells revealed a significant increase in EdU incorporation in the mCherry$^+$ population following *Ovol1* knockdown, while EdU incorporation in the mCherry$^-$ population remained unchanged (Figure 5C). Therefore, reprogramming intermediates display improved proliferation when OVOL1 is suppressed.

In the low-KLF4 condition, mCherry$^+$ cells show minimal induction of transient MET genes and proliferate rapidly resulting in a diminished proportion of Nanog-GFP$^+$ iPSCs on day 18 (Figures 1A–1D). We addressed whether the expression of OVOL1 could suppress the expansion of mCherry$^+$ cells, and possibly enhance the efficiency of low-KLF4 reprogramming (Figure 5D). Quantification of whole-well images revealed that *Ovol1* overexpression in the low-KLF4 condition significantly reduced total colony numbers, while overexpression in the high-KLF4 condition had only a minor effect (Figures 5E and 5F). In both cases, the number of Nanog-GFP$^+$ colonies was proportionally reduced, as confirmed by flow cytometry (Figure 5G). These results indicate that while OVOL1 can suppress proliferation of failed reprogramming cells, its overexpression has an indiscriminate effect on all reprogramming populations and does not directly support the acquisition of pluripotency.

DISCUSSION

Somatic cell reprogramming processes diverge depending on the factor stoichiometry established by each reprogramming method (Chantzoura et al., 2015; Kim et al., 2015). Carey et al. (2011) first recognized phenotypic differences between OSKM and STEMCCA polycistronic cassettes, which could be rescued by supplementation of STEMCCA with additional OCT3/4 and KLF4. Our results reiterate the importance of reprogramming factor stoichiometry in order to accurately interpret reprogramming processes and outcomes. In addition to the sustained “core epithelial genes,” the intermediates in high-KLF4 reprogramming express transient “peripheral epithelial genes” such as the transcriptional regulator *Ovol1* and cell-surface protein *Tacstd2*. Importantly, the transient upregulation of *Tacstd2* and *Ovol1* were commonly observed in various high-KLF4 reprogramming systems (e.g., MKOS and OSKM).

Intermediate states during reprogramming have recently regained attention as their influence on reprogramming outcomes are becoming clearer. Studies exploring chromatin structure and Yamanaka factor binding suggest somatic enhancer silencing and the formation of transiently opened chromatin regions (Chronis et al., 2017; Knaupp et al., 2017; Li et al., 2017). These data show that KLF4 does not occupy *Ovol1* or *Tacstd2* in MEF early reprogramming (48 hr) or mESCs (Chronis et al., 2017), times at which peripheral MET gene expression is not detected in

(F) Quantification of Nanog-GFP$^-$ and Nanog-GFP$^+$ colony numbers on day 18 following overexpression of *Ovol1* in low- and high-KLF4. Means ± SD for total colonies from three independent experiments (Student’s t test, *Ovol1* versus mock in low-KLF4, $p = 0.0063$; *Ovol1* versus mock in high-KLF4, $p = 0.052$). $^{**}p < 0.01$.
(G) Flow-cytometry analysis on day 18 for Nanog-GFP and mCherry following overexpression of *Ovol1* in low- and high-KLF4.

our system. However, in one pre-iPSC line, KLF4 binds *Ovol1* at the promoter and near exon 2, the site of a strong ATAC-seq (assay for transposase-accessible chromatin using sequencing) signal. Neither OCT3/4 nor SOX2 are ever found to occupy *Ovol1* (Chronis et al., 2017; Knaupp et al., 2017), suggesting that KLF4 may directly regulate *Ovol1* expression. Compellingly, the same pre-iPSC sample shows open chromatin at the promoters of various peripheral MET genes including *Tacstd2*, which is not occupied by OCT3/4, SOX2, or KLF4 (Chronis et al., 2017). Further experiments will be required to determine the genetic hierarchy and regulation of these intermediate cell states.

Based on the transient induction of MET genes and highly efficient reprogramming in high-KLF4, we initially hypothesized that aspects of transient MET may promote the reprogramming process. However, the transient MET genes interrogated in this study by CRISPR-interference repression or overexpression of OVOL1 appear to be unnecessary for pluripotency acquisition. Moreover, suppression of OVOL1 in high-KLF4 reprogramming not only resulted in diminished transient MET gene expression, but in turn promoted the expansion of mCherry^{+} intermediate cells by derepression of cell proliferation. These results are consistent with OVOL1 biological functions regulating epidermal differentiation and proliferation (Lee et al., 2014; Nair et al., 2006). Thus, we conclude that early activation of OVOL1 is responsible for the characteristically subdued induction phase and preventing the expansion of failed reprogramming cells by trapping them in an alternative epithelial state of low proliferation. As a result, OVOL1 contributes positively to the uniformity of Nanog-GFP^{+} iPSC emergence and apparently potent reprogramming capacity of the high-KLF4 condition.

Precise enrichment of specific cell populations is required to reveal the underlying molecular aspects of deterministic events during reprogramming. In this study, we revealed that under high-KLF4 conditions the majority of cells that become iPSCs follow the same cell-surface marker dynamics from TROP2^{+}SSEA-1^{-} to TROP2^{+}SSEA-1^{+}, and finally TROP2^{-}SSEA-1^{+}. Since the reprogramming capacities of each population are correlated with the temporal order of marker presentation, TROP2 activation and repression either indicates a required step in early reprogramming or escape from a dominant and competing reprogramming pathway. Based on the reduced reprogramming potential of day 14 TROP2^{+} cells, we propose that the progression from a transient MET population to an early pluripotent state acts as an important and previously undescribed bottleneck in reprogramming. The combination of TROP2 and SSEA-1 can be applied as a population-specific enrichment method for further molecular analysis.

In summary, we reveal that transient MET induction regulated by OVOL1 is not necessary for the acquisition of pluripotency, but rather plays an important role in suppressing the expansion of failed reprogramming of cells by trapping them in an alternative epithelial state. Our study comparing two disparate KLF4 stoichiometries provides new insights into how intermediate reprogramming states ultimately direct cell-fate decisions.

EXPERIMENTAL PROCEDURES

Full experimental procedures and associated references are available in Supplemental Experimental Procedures.

Plasmid Construction

A list of sequence-verified plasmids and primers used for cloning is provided in Table S4. Complete sequences are available through Addgene (plasmid numbers 120352–120360) or upon request.

MEF Isolation and PB Reprogramming

MEFs were isolated and reprogramming was induced as described previously (Woltjen et al., 2016). Animal care and experiments using animal tissues and primary cell cultures were approved by the CiRA Animal Experiment Committee in accordance with Kyoto University guidelines. Different amounts of transposons were utilized to achieve similar transfection efficiencies based on mCherry induction (500 ng: PB-TAC-OKMS and -OK^{+9}MS, PB-U6-sgRNA, PB-TAB-*LacZ*, and -*Ovol1*; 1,500 ng: PB-TAC-OKMS-Cas9, -OKMS-KRAB-dCas9, OK^{+9}MS-Cas9 and -OK^{+9}MS-KRAB-dCas9). One thousand nanograms of pCyL43 PB transposase plasmid was used regardless of the total transposon amount.

Whole-Well Fluorescence Microscopy Imaging

Images were acquired with a Nikon BioStation CT (Nikon) equipped with GFP and mCherry fluorescence filters and phase contrast using 2× lenses. Colony count analysis was performed using a custom macro for CL-Quant 3.0. The threshold parameters were set by adding 5 times the SDs to the mean intensities of GFP-negative or mCherry-negative colonies in the background-subtracted images.

Microarray Analysis

RNA isolation, data acquisition, and data processing were performed as described by Kim et al. (2015). The averages of two independent experiments were used for the following samples: mESCs, day 8 mCherry^{+} intermediate reprogramming populations (OKMS and OK^{+9}MS), primary keratinocytes, *Klf4* mCherry^{+}, and day 6 *Klf4*$^{+9}$ mCherry^{+} intermediate reprogramming population. Microarray data for day 6 reprogramming intermediates and day 2 MEF (Mock) were previously described and deposited in the Gene Expression Omnibus (GEO) under accession number GEO: GSE65468 (Kim et al., 2015). All additional time points are available under accession number GEO: GSE116309.

Statistical Analysis

The data are presented as the means ± SD from indicated numbers of independent experiments. Student's t tests for detecting significance of biological difference were used for all statistical analysis.

ACCESSION NUMBERS

Deposited microarray data can be found in the GEO under accession number GEO: GSE116309.

SUPPLEMENTAL INFORMATION

Supplemental Information includes Supplemental Experimental Procedures, five figures, four tables, and one video and can be found with this article online at https://doi.org/10.1016/j.stemcr.2018.12.008.

AUTHOR CONTRIBUTIONS

H.K. and K.W. conceived the study. H.K., R.S., T.Y., T.S., and K.W. designed experiments and interpreted the data. H.K. performed the majority of experiments. S.-I.K. performed mCherry cell sorting for microarray analysis. R.S. performed live cell staining with time-lapse imaging. F.O.-Y. developed the colony-counting macro and isolated primary keratinocytes. H.K. performed bioinformatics analyses with advice from T.Y. The manuscript was written by H.K. and K.W. with input from all authors.

ACKNOWLEDGMENTS

We would like to acknowledge the technical assistance of Ryoko Hirohata for plasmid construction, Michiko Nakamura and Toshiko Sato for gene expression analysis by qRT-PCR and microarray, and Kanae Mitsunaga for cell sorting. We extend our gratitude to Bruce Conklin for providing the KRAB-Cas9 plasmid, as well as Feng Zhang for provision of plasmids through Addgene. This work was funded in part by grants to K.W. from AMED (nos. JP17jm0210039 and JP18bm0104001) and to T.S. from the National Science Foundation (no. 31003A_156431). H.K. is supported by a Grant-in-Aid for JSPS Research Fellows (JP18J14431), and R.S. by the Kyoto University Foundation Long-Term Fellowship for Study Abroad.

Received: September 6, 2018
Revised: December 11, 2018
Accepted: December 12, 2018
Published: January 10, 2019

REFERENCES

Albino, D., Longoni, N., Curti, L., Mello-Grand, M., Pinton, S., Civenni, G., Thalmann, G., D'Ambrosio, G., Sarti, M., Sessa, F., et al. (2012). ESE3/EHF controls epithelial cell differentiation and its loss leads to prostate tumors with mesenchymal and stem-like features. Cancer Res. *72*, 2889–2900.

Andrews, P.W. (2011). Toward safer regenerative medicine. Nat. Biotechnol. *29*, 803–805.

Brinkman, E.K., Chen, T., Amendola, M., and van Steensel, B. (2014). Easy quantitative assessment of genome editing by sequence trace decomposition. Nucleic Acids Res. *42*, e168.

Buganim, Y., Faddah, D.A., Cheng, A.W., Itskovich, E., Markoulaki, S., Ganz, K., Klemm, S.L., van Oudenaarden, A., and Jaenisch, R. (2012). Single-cell expression analyses during cellular reprogramming reveal an early stochastic and a late hierarchic phase. Cell *150*, 1209–1222.

Cacchiarelli, D., Trapnell, C., Ziller, M.J., Soumillon, M., Cesana, M., Karnik, R., Donaghey, J., Smith, Z.D., Ratanasirintrawoot, S., Zhang, X., et al. (2015). Integrative analyses of human reprogramming reveal dynamic nature of induced pluripotency. Cell *162*, 412–424.

Carey, B.W., Markoulaki, S., Hanna, J., Saha, K., Gao, Q., Mitalipova, M., and Jaenisch, R. (2009). Reprogramming of murine and human somatic cells using a single polycistronic vector. Proc. Natl. Acad. Sci. U S A *106*, 157–162.

Carey, B.W., Markoulaki, S., Hanna, J.H., Faddah, D.A., Buganim, Y., Kim, J., Ganz, K., Steine, E.J., Cassady, J.P., Creyghton, M.P., et al. (2011). Reprogramming factor stoichiometry influences the epigenetic state and biological properties of induced pluripotent stem cells. Cell Stem Cell *9*, 588–598.

Chakrabarti, R., Hwang, J., Andres Blanco, M., Wei, Y., Lukacisin, M., Romano, R.A., Smalley, K., Liu, S., Yang, Q., Ibrahim, T., et al. (2012). Elf5 inhibits the epithelial-mesenchymal transition in mammary gland development and breast cancer metastasis by transcriptionally repressing Snail2. Nat. Cell Biol. *14*, 1212–1222.

Chantzoura, E., Skylaki, S., Menendez, S., Kim, S.I., Johnsson, A., Linnarsson, S., Woltjen, K., Chambers, I., and Kaji, K. (2015). Reprogramming roadblocks are system dependent. Stem Cell Reports *5*, 350–364.

Chevillard, G., and Blank, V. (2011). NFE2L3 (NRF3): the Cinderella of the Cap'n'Collar transcription factors. Cell. Mol. Life Sci. *68*, 3337–3348.

Chou, B.K., Mali, P., Huang, X., Ye, Z., Dowey, S.N., Resar, L.M., Zou, C., Zhang, Y.A., Tong, J., and Cheng, L. (2011). Efficient human iPS cell derivation by a non-integrating plasmid from blood cells with unique epigenetic and gene expression signatures. Cell Res. *21*, 518–529.

Chronis, C., Fiziev, P., Papp, B., Butz, S., Bonora, G., Sabri, S., Ernst, J., and Plath, K. (2017). Cooperative binding of transcription factors orchestrates reprogramming. Cell *168*, 442–459.e20.

David, L., and Polo, J.M. (2014). Phases of reprogramming. Stem Cell Res. *12*, 754–761.

Hong, H., Takahashi, K., Ichisaka, T., Aoi, T., Kanagawa, O., Nakagawa, M., Okita, K., and Yamanaka, S. (2009). Suppression of induced pluripotent stem cell generation by the p53-p21 pathway. Nature *460*, 1132–1135.

Horlbeck, M.A., Gilbert, L.A., Villalta, J.E., Adamson, B., Pak, R.A., Chen, Y., Fields, A.P., Park, C.Y., Corn, J.E., Kampmann, M., et al. (2016). Compact and highly active next-generation libraries for CRISPR-mediated gene repression and activation. Elife *5*. https://doi.org/10.7554/eLife.19760.

Kaji, K., Norrby, K., Paca, A., Mileikovsky, M., Mohseni, P., and Woltjen, K. (2009). Virus-free induction of pluripotency and subsequent excision of reprogramming factors. Nature *458*, 771–775.

Kim, S.I., Oceguera-Yanez, F., Hirohata, R., Linker, S., Okita, K., Yamada, Y., Yamamoto, T., Yamanaka, S., and Woltjen, K. (2015). KLF4 N-terminal variance modulates induced reprogramming to pluripotency. Stem Cell Reports *4*, 727–743.

Knaupp, A.S., Buckberry, S., Pflueger, J., Lim, S.M., Ford, E., Larcombe, M.R., Rossello, F.J., de Mendoza, A., Alaei, S., Firas, J., et al. (2017). Transient and permanent reconfiguration of chromatin and transcription factor occupancy drive reprogramming. Cell Stem Cell *21*, 834–845.e6.

Koike-Yusa, H., Li, Y., Tan, E.P., Velasco-Herrera Mdel, C., and Yusa, K. (2014). Genome-wide recessive genetic screening in mammalian cells with a lentiviral CRISPR-guide RNA library. Nat. Biotechnol. *32*, 267–273.

Kuan, I.I., Liang, K.H., Wang, Y.P., Kuo, T.W., Meir, Y.J., Wu, S.C., Yang, S.C., Lu, J., and Wu, H.C. (2017). EpEX/EpCAM and Oct4 or Klf4 alone are sufficient to generate induced pluripotent stem cells through STAT3 and HIF2alpha. Sci. Rep. *7*, 41852.

Kudo, T., Kaneko, M., Iwasaki, H., Togayachi, A., Nishihara, S., Abe, K., and Narimatsu, H. (2004). Normal embryonic and germ cell development in mice lacking alpha 1,3-fucosyltransferase IX (Fut9) which show disappearance of stage-specific embryonic antigen 1. Mol. Cell. Biol. *24*, 4221–4228.

Lee, B., Villarreal-Ponce, A., Fallahi, M., Ovadia, J., Sun, P., Yu, Q.C., Ito, S., Sinha, S., Nie, Q., and Dai, X. (2014). Transcriptional mechanisms link epithelial plasticity to adhesion and differentiation of epidermal progenitor cells. Dev. Cell *29*, 47–58.

Li, D., Liu, J., Yang, X., Zhou, C., Guo, J., Wu, C., Qin, Y., Guo, L., He, J., Yu, S., et al. (2017). Chromatin accessibility dynamics during iPSC reprogramming. Cell Stem Cell *21*, 819–833.e6.

Li, R., Liang, J., Ni, S., Zhou, T., Qing, X., Li, H., He, W., Chen, J., Li, F., Zhuang, Q., et al. (2010). A mesenchymal-to-epithelial transition initiates and is required for the nuclear reprogramming of mouse fibroblasts. Cell Stem Cell *7*, 51–63.

Lujan, E., Zunder, E.R., Ng, Y.H., Goronzy, I.N., Nolan, G.P., and Wernig, M. (2015). Early reprogramming regulators identified by prospective isolation and mass cytometry. Nature *521*, 352–356.

Mandegar, M.A., Huebsch, N., Frolov, E.B., Shin, E., Truong, A., Olvera, M.P., Chan, A.H., Miyaoka, Y., Holmes, K., Spencer, C.I., et al. (2016). CRISPR interference efficiently induces specific and reversible gene silencing in human iPSCs. Cell Stem Cell *18*, 541–553.

McDougall, A.R., Tolcos, M., Hooper, S.B., Cole, T.J., and Wallace, M.J. (2015). Trop2: from development to disease. Dev. Dyn. *244*, 99–109.

Nair, M., Bilanchone, V., Ortt, K., Sinha, S., and Dai, X. (2007). Ovol1 represses its own transcription by competing with transcription activator c-Myb and by recruiting histone deacetylase activity. Nucleic Acids Res. *35*, 1687–1697.

Nair, M., Teng, A., Bilanchone, V., Agrawal, A., Li, B., and Dai, X. (2006). Ovol1 regulates the growth arrest of embryonic epidermal progenitor cells and represses c-myc transcription. J. Cell Biol. *173*, 253–264.

Nefzger, C.M., Rossello, F.J., Chen, J., Liu, X., Knaupp, A.S., Firas, J., Paynter, J.M., Pflueger, J., Buckberry, S., Lim, S.M., et al. (2017). Cell type of origin dictates the route to pluripotency. Cell Rep. *21*, 2649–2660.

O'Malley, J., Skylaki, S., Iwabuchi, K.A., Chantzoura, E., Ruetz, T., Johnsson, A., Tomlinson, S.R., Linnarsson, S., and Kaji, K. (2013). High-resolution analysis with novel cell-surface markers identifies routes to iPS cells. Nature *499*, 88–91.

Polo, J.M., Anderssen, E., Walsh, R.M., Schwarz, B.A., Nefzger, C.M., Lim, S.M., Borkent, M., Apostolou, E., Alaei, S., Cloutier, J., et al. (2012). A molecular roadmap of reprogramming somatic cells into iPS cells. Cell *151*, 1617–1632.

Richardson, R.J., Dixon, J., Malhotra, S., Hardman, M.J., Knowles, L., Boot-Handford, R.P., Shore, P., Whitmarsh, A., and Dixon, M.J. (2006). Irf6 is a key determinant of the keratinocyte proliferation-differentiation switch. Nat. Genet. *38*, 1329–1334.

Roca, H., Hernandez, J., Weidner, S., McEachin, R.C., Fuller, D., Sud, S., Schumann, T., Wilkinson, J.E., Zaslavsky, A., Li, H., et al. (2013). Transcription factors OVOL1 and OVOL2 induce the mesenchymal to epithelial transition in human cancer. PLoS One *8*, e76773.

Samavarchi-Tehrani, P., Golipour, A., David, L., Sung, H.K., Beyer, T.A., Datti, A., Woltjen, K., Nagy, A., and Wrana, J.L. (2010). Functional genomics reveals a BMP-driven mesenchymal-to-epithelial transition in the initiation of somatic cell reprogramming. Cell Stem Cell *7*, 64–77.

Sommer, C.A., Stadtfeld, M., Murphy, G.J., Hochedlinger, K., Kotton, D.N., and Mostoslavsky, G. (2009). Induced pluripotent stem cell generation using a single lentiviral stem cell cassette. Stem Cells *27*, 543–549.

Sridharan, R., Tchieu, J., Mason, M.J., Yachechko, R., Kuoy, E., Horvath, S., Zhou, Q., and Plath, K. (2009). Role of the murine reprogramming factors in the induction of pluripotency. Cell *136*, 364–377.

Takada, Y., Ye, X., and Simon, S. (2007). The integrins. Genome Biol. *8*, 215.

Takahashi, K., Tanabe, K., Ohnuki, M., Narita, M., Sasaki, A., Yamamoto, M., Nakamura, M., Sutou, K., Osafune, K., and Yamanaka, S. (2014). Induction of pluripotency in human somatic cells via a transient state resembling primitive streak-like mesendoderm. Nat. Commun. *5*, 3678.

Takahashi, K., and Yamanaka, S. (2006). Induction of pluripotent stem cells from mouse embryonic and adult fibroblast cultures by defined factors. Cell *126*, 663–676.

Werner, S., Beer, H.D., Mauch, C., Luscher, B., and Werner, S. (2001). The Mad1 transcription factor is a novel target of activin and TGF-beta action in keratinocytes: possible role of Mad1 in wound repair and psoriasis. Oncogene *20*, 7494–7504.

Woltjen, K., Kim, S.I., and Nagy, A. (2016). The piggyBac transposon as a platform technology for somatic cell reprogramming studies in mouse. Methods Mol. Biol. *1357*, 1–22.

Yusa, K., Rad, R., Takeda, J., and Bradley, A. (2009). Generation of transgene-free induced pluripotent mouse stem cells by the piggyBac transposon. Nat. Methods *6*, 363–369.

Zhang, H., Pasolli, H.A., and Fuchs, E. (2011). Yes-associated protein (YAP) transcriptional coactivator functions in balancing growth and differentiation in skin. Proc. Natl. Acad. Sci. U S A *108*, 2270–2275.

Zunder, E.R., Lujan, E., Goltsev, Y., Wernig, M., and Nolan, G.P. (2015). A continuous molecular roadmap to iPSC reprogramming through progression analysis of single-cell mass cytometry. Cell Stem Cell *16*, 323–337.

Stem Cell Reports, Volume *12*

Supplemental Information

OVOL1 Influences the Determination and Expansion of iPSC Reprogramming Intermediates

Harunobu Kagawa, Ren Shimamoto, Shin-Il Kim, Fabian Oceguera-Yanez, Takuya Yamamoto, Timm Schroeder, and Knut Woltjen

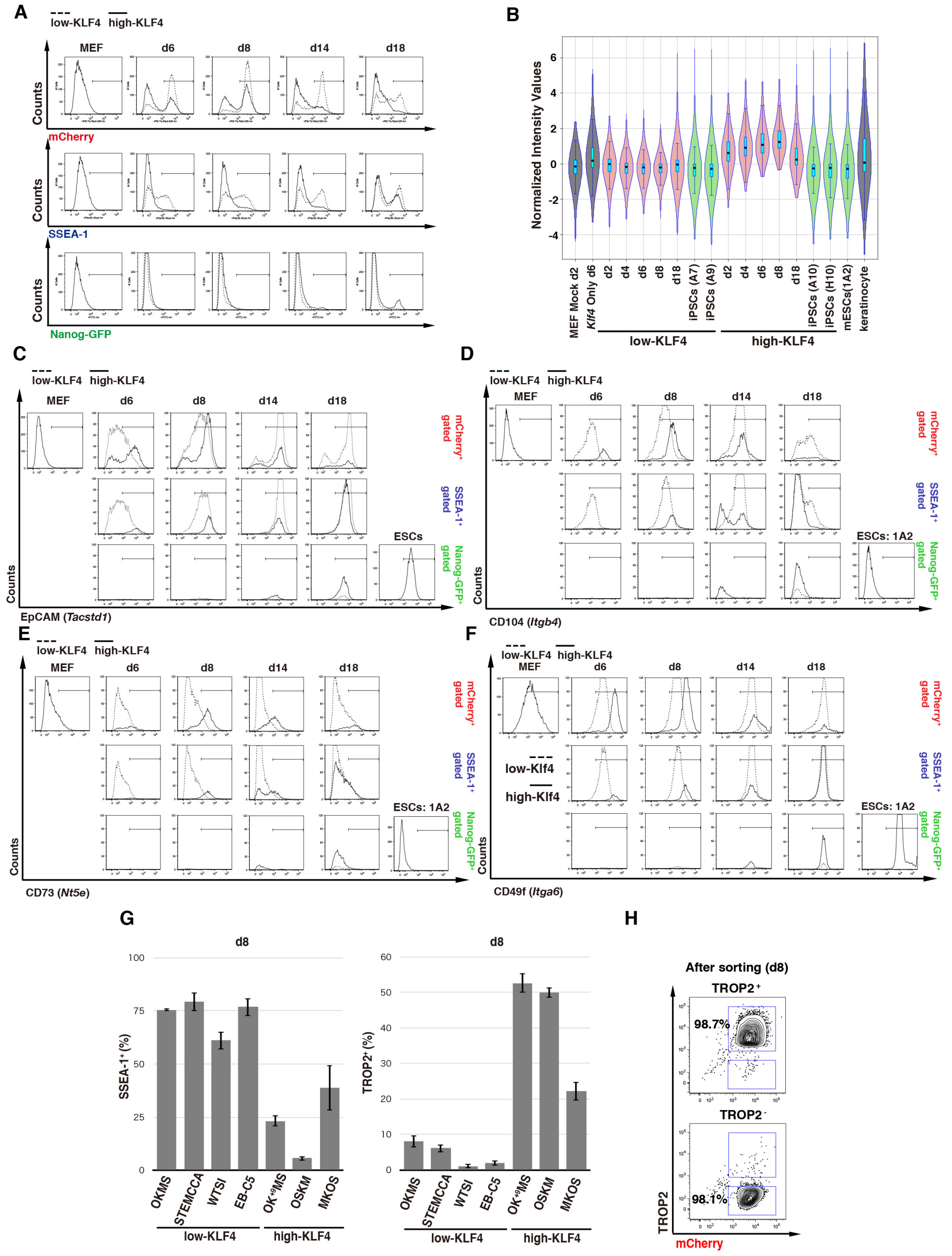

Figure S1. Characterizing low- and high-KLF4 mediated reprogramming

(A) Flow cytometry analysis for mCherry, SSEA-1, and Nanog-GFP during low- and high-KLF4 MEF reprogramming.

(B) Violin plot of normalized intensity values for high-KLF4 specific genes in low- or high-KLF4 reprogramming processes. The box plot displays the median, 25th, and 75th percentiles.

(C-F) FACS analysis of cell surface marker dynamics. Histograms are grouped by analysis day (columns) or population gating (rows). Dashed lines and straight lines represent low-KLF4 and high-KLF4, respectively.

(G) Proportion of SSEA-1^{+} (left) and TROP2^{+} (right) on d8 in publically available low- and high-KLF4 reprogramming systems (Low: OKMS, STEMCCA, WTSI and EB-C5; High: OK^{+9}MS, OSKM and MKOS). Means ± SD for three independent experiments.
(H) FACS plots verifying sorting efficiency for TROP2^{-} and TROP2^{+} populations at d8 in high-KLF4.

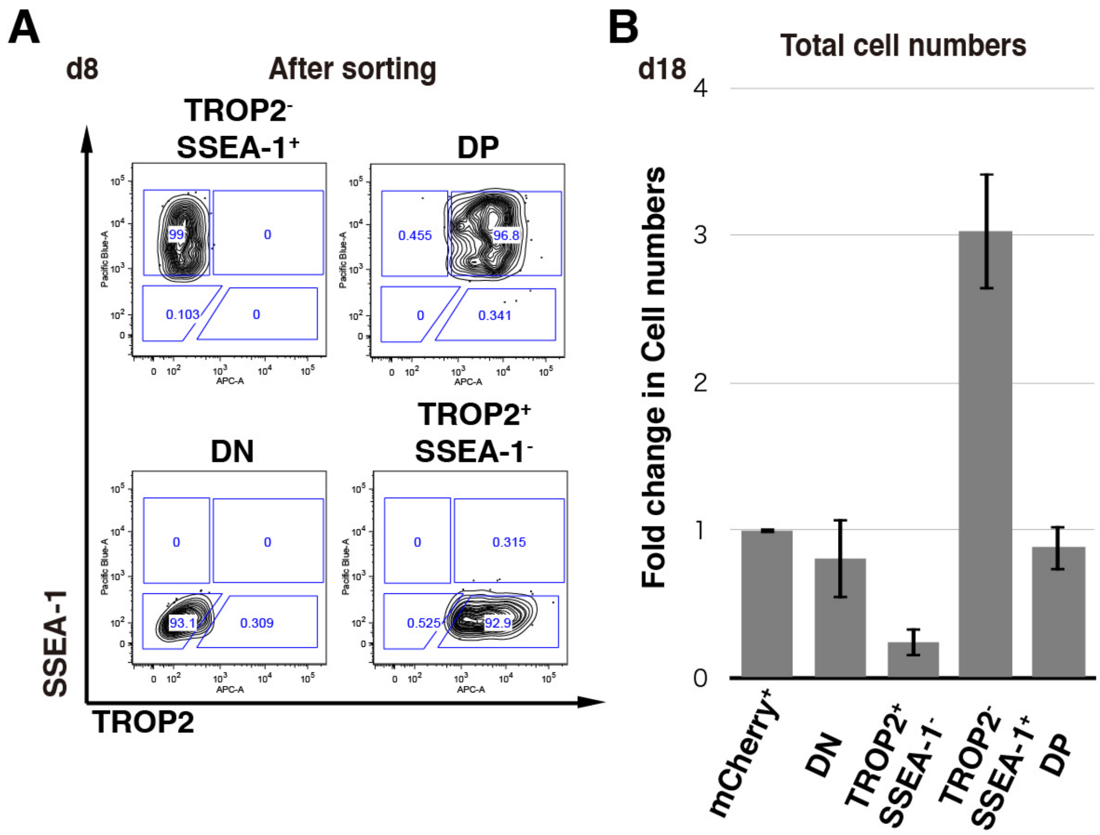

Figure S2. Reprogramming capacity of TROP2 and SSEA-1 marked populations

(A) FACS plots verifying sorting efficiency for double negative (DN), TROP2^{+}SSEA-1^{-}, TROP2^{-}SSEA-1^{+}, and double positive (DP) populations at d8 in high-KLF4.

(B) Total cell numbers on d18 following d8 sorting based on TROP2 and SSEA-1 presentation. Total cell numbers are normalized to mCherry^{+}. Means ± SD for three independent experiments (n=3).

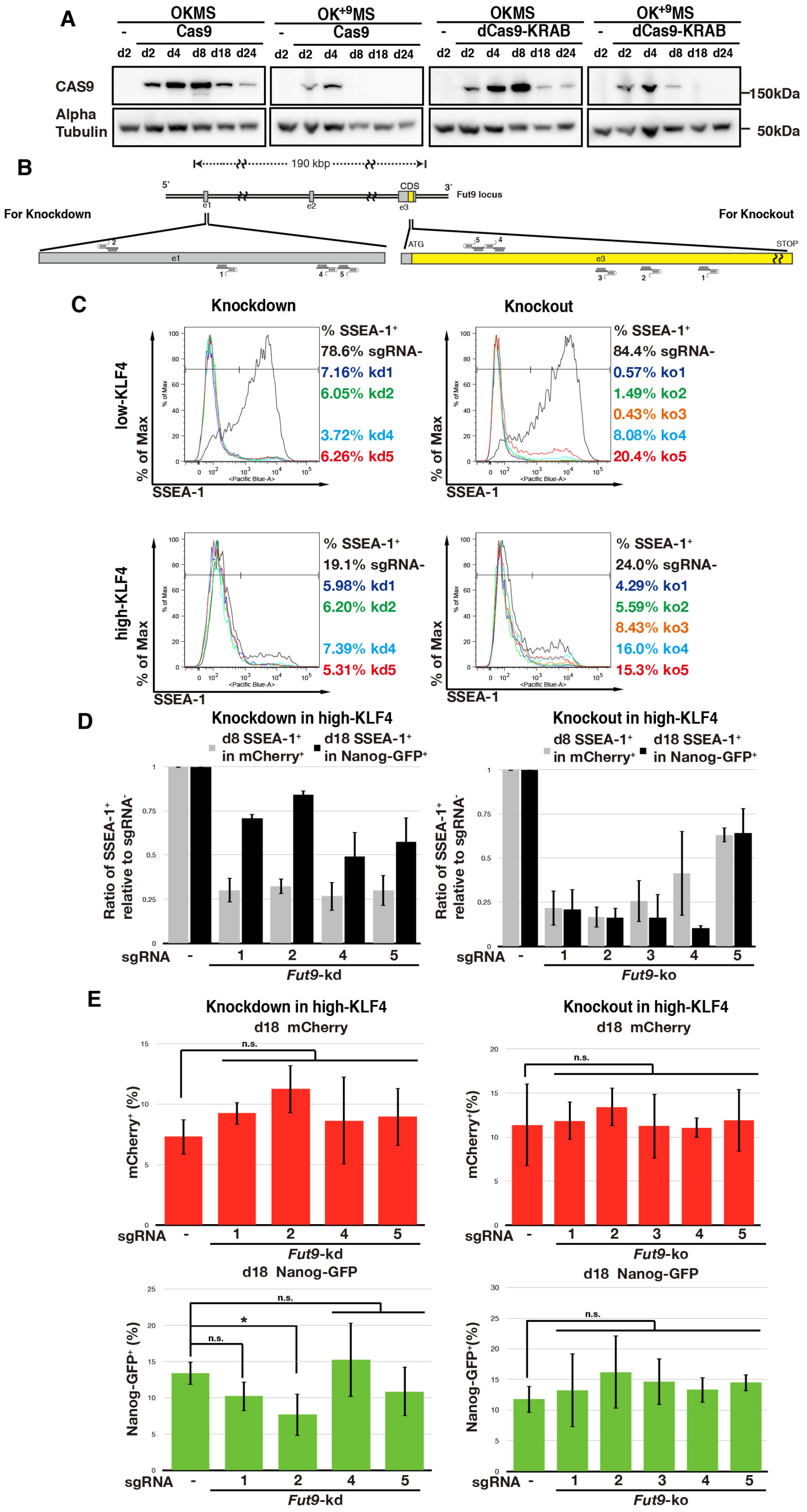

Figure S3. Establishment of CRISPR/Cas9 knockdown and knockout systems during reprogramming

(A) Western blot analysis of CAS9 and KRAB-dCas9 expression in low- and high-KLF4 from d2 – d24. Alpha Tubulin was used as a loading control.

(B) Schematic diagrams of the *Fut9* locus (NC_000070.6, 25609333..25800003, complement) with detailed segments of exon 1 and exon 3 to show sgRNA target sites.

(C) *Fut9* knockdown (left) and knockout (right) efficiencies by flow cytometry. Histograms depict the SSEA-1 presentation at d8 in low-KLF4 (top) and high-KLF4 (bottom).

(D) SSEA-1 presentation following *Fut9* knockdown (left) and knockout (right) in high-KLF4 detected by flow cytometry at d8 in the $mCherry^+$ population and at d18 in the $Nanog\text{-}GFP^+$ population. Bar graphs depict the normalized percentage of $SSEA\text{-}1^+$ population to sgRNA-. Means ± SD for three independent experiments.

(E) Proportion of $mCherry^+$ (top) and $Nanog\text{-}GFP^+$ (bottom) on d18 following *Fut9* knockdown (left) and knockout (right) in high-KLF4. Only *Fut9*-kd2 presented a sigfinificant difference in acquisition of Nanog-GFP. Means ± SD for three independent experiments. *, $p<0.05$. Student t-test

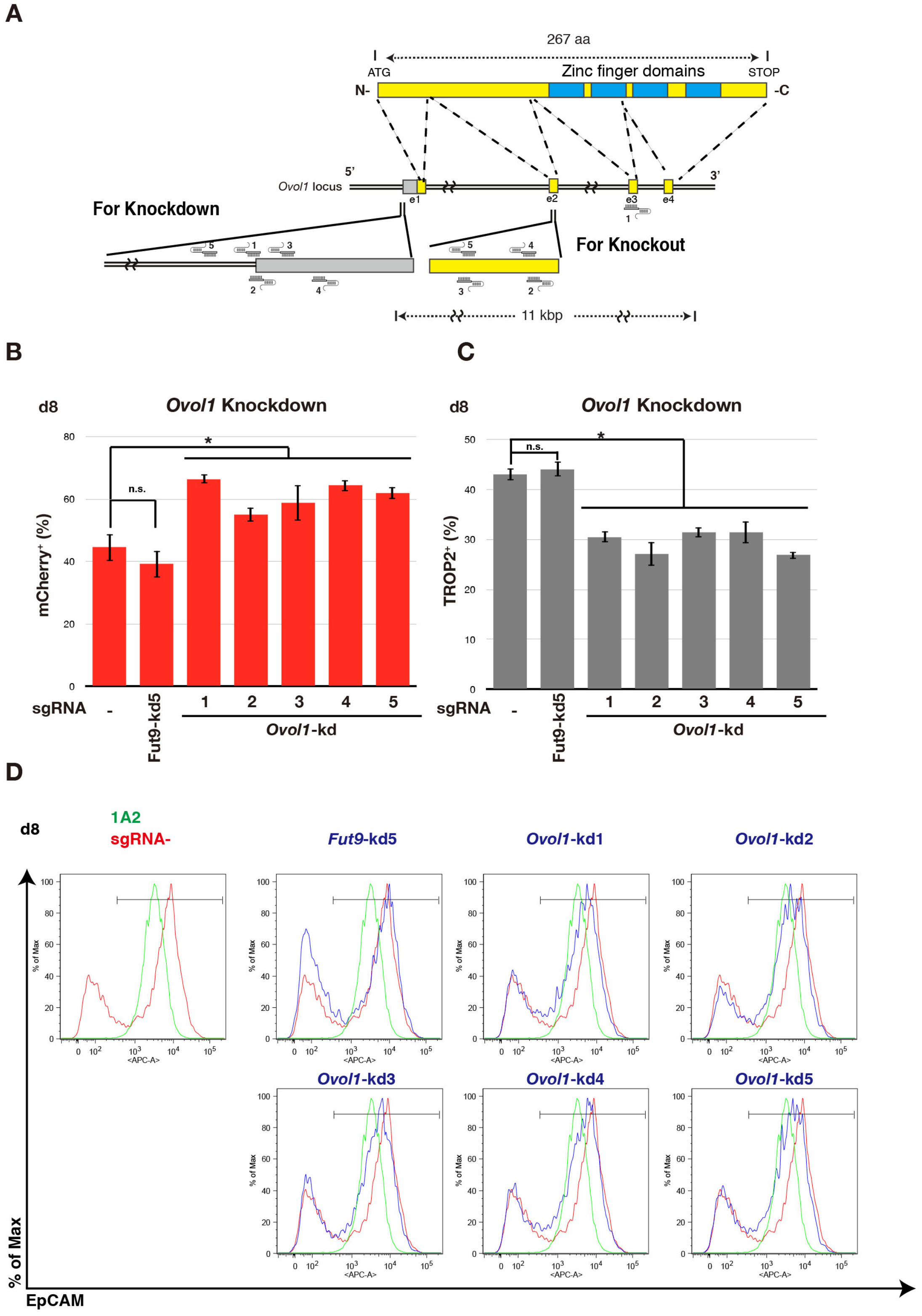

Figure S4. Cas9 and KRAB-Cas9 sgRNA design and validation for *Ovol1*

(A) Schematic diagram showing the *Ovol1* locus (NC_000085.6, 5549137..5560575, complement) with detailed segments of exon 1 and exon 2 to show sgRNA target sites. The OVOL1 protein (UniProt: Q9WTJ2), including the zinc finger domains, is shown above.

(B) Proportion of $mCherry^+$ on d8 in high-KLF4 reprogramming with or without *Ovol1* knockdown. Means ± SD for five independent experiments. *, $p<0.05$. Student t-test

(C) Proportion of $TROP2^+$ on d8 in high-KLF4 reprogramming with or without *Ovol1* knockdown. Means ± SD for three independent experiments. *, $p<0.05$. Student t-test

(D) FACS analysis of EpCAM expression dynamics. Each knockdown sample is presented by blue lines. Green lines and red lines represent 1A2 and sgRNA-, respectively.

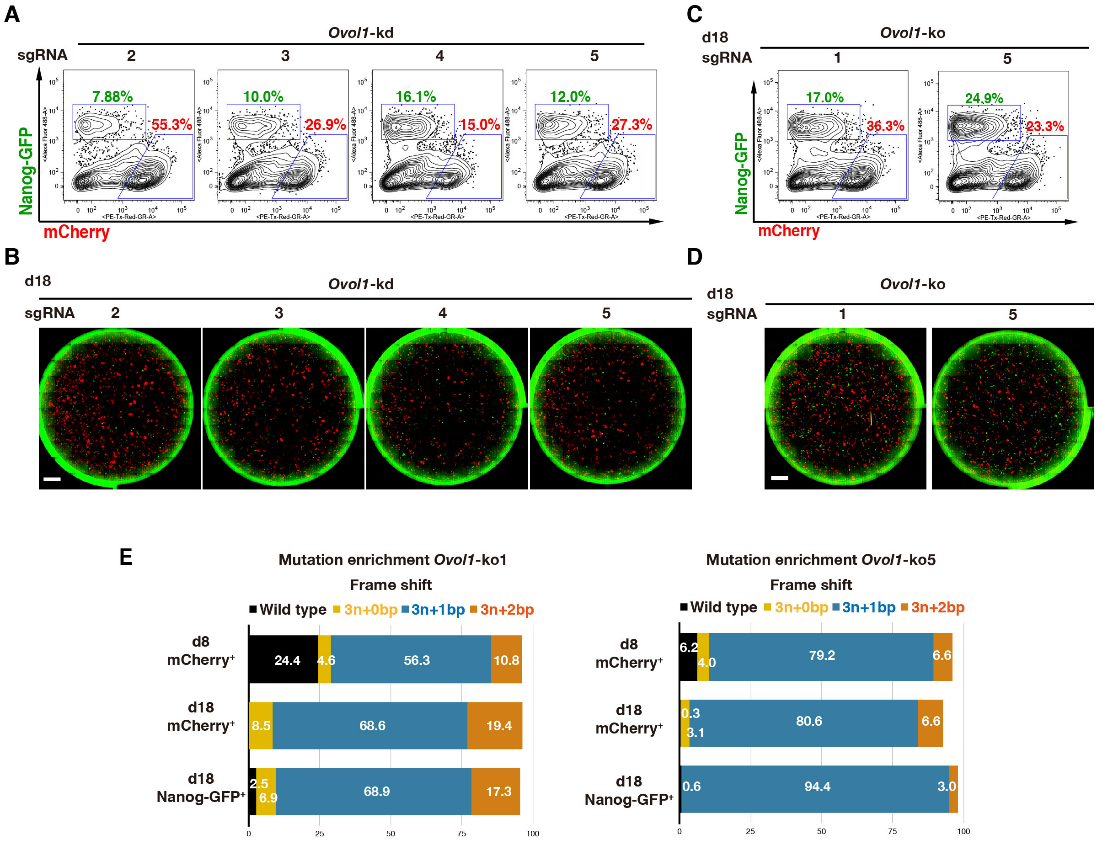

Figure S5. Effects of *Ovol1* knockdown or knockout on reprogramming efficiency

(A) FACS analysis of Nanog-GFP and mCherry on d18 following *Ovol1* knockdown in high-KLF4.

(B) Whole-well fluorescence microscopy images for Nanog-GFP and mCherry on d18 following *Ovol1* knockdown in high-KLF4. Scale bars, 4,000 µm.

(C) FACS analysis of Nanog-GFP and mCherry on d18 following *Ovol1* knockout in high-KLF4.

(D) Whole-well fluorescence microscopy images for Nanog-GFP and mCherry on d18 following *Ovol1* knockout in high-KLF4. Scale bars, 4,000 µm.

(E) Distribution of mutation frequencies predicted by TIDE in d8 mCherry^{+}, d18 mCherry^{+} and d18 Nanog-GFP^{+} populations following *Ovol1* knockout in high-KLF4. Mutant alleles were categorized based on a resulting in-frame indels 3n + 0 bp (yellow), frame-shifted indels 3n + 1 bp (blue), or 3n + 2 bp (orange). Wild type (black) alleles showed no indel. Data are representative of two independent experiments.

Supplemental Experimental Procedures

Plasmid Construction

A list of sequence-verified plasmids and primers used for cloning is listed in Table S4. Complete sequences are available through Addgene (plasmid #'s 120352-120360) or upon request. Detailed cloning histories are available upon request as Snapgene files. Briefly, PB-TAC-Cas9 plasmid were generated by standard cloning using restriction digestion and ligation. The Cas9 cassette was cloned from pX330-U6-Chimeric_BB-CBh-hSpCas9 provided by Feng Zhang (Addgene plasmid # 42230). The plasmid PB-TAC-KRAB-dCas9 was generated by PCR amplification, restriction digestion and ligation. KRAB-dCas9 was cloned from pUCM-AAV-NC-TO-KC provided by Bruce Conklin (Mandegar et al., 2016). Yamanaka factors were inserted into these plasmids by restriction digestion and ligation or by Gateway LR clonase reaction (Invitrogen, Cat.11791100) as described previously (Woltjen et al., 2009). For sgRNA expression, PB-U6 plasmid was generated by restriction digestion and ligation. The sgRNA scaffold was cloned from pX330-U6-Chimeric_BB-CBh-hSpCas9. Oligos encoding sgRNA were annealed and cloned into PB-U6 linearized with BbsI as previously described (Ran et al., 2013). The resulting plasmids were sequence verified using primer dna790. sgRNA identification numbers (ie. kd1-5 and ko1-5) are ordered based on the predicted activity score from previously published sgRNA libraries (Horlbeck et al., 2016; Koike-Yusa et al., 2014). For complementation experiments, a synthesized DNA fragment composed of the *Ovol1* cDNA sequence flanked by attB1 and attB2 sites (IDT) was cloned into pDONR221 using Gateway BP clonase reaction (Invitrogen Cat.11789020). PB-TAB vectors containing *Ovol1* or *LacZ* (mock) were generated by Gateway LR clonase reaction.

MEF Isolation and PB Reprogramming

MEFs were isolated from E13.5 mouse embryos resulting from the mating of homozygous Nanog-GFP (Okita et al., 2007) transgenic males and homozygous ROSA26-rtTA (Ohnishi et al., 2014) transgenic females on a C57BL/6 background, or wild-type C57BL/6 mice (without ROSA26-rtTA or Nanog-GFP transgenes), and cultured as described previously (Woltjen et al., 2016). Animal care and experiments using animal tissues and primary cell culture were approved by the CiRA Animal Experiment Committee in accordance with Kyoto University guidelines. MEFs were seeded in DMEM containing 10% FBS, penicillin-streptomycin, and L-glutamine on gelatin coated 6-well dishes at a density of 1×10^5 cells per well. After a 24 hr culture, FugeneHD (Promega, Cat.E2312) was used to transfect cells at a Fugene/DNA ratio of 4:1. Different amounts of transposons were utilized in transfection to achieve similar transfection efficiencies based on mCherry induction (500 ng: PB-TAC-OKMS and -OK^{+9}MS, PB-U6-sgRNA, PB-TAB-*LacZ*, and -*Ovol1*, 1,500 ng: PB-TAC-OKMS-Cas9, -OKMS-KRAB-dCas9, OK^{+9}MS-Cas9 and -OK^{+9}MS-KRAB-dCas9). 1,000 ng of pCyL43 PB transposase plasmid was used regardless of the total amount of transposons. After 24 hr, the medium was replaced with ESC medium (DMEM containing 15% FBS, penicillin-streptomycin, GlutaMAX, β-mercaptoethanol, sodium-pyruvate, non-essential amino acids, LIF, and dox [1 μg/mL]). After transfection, cells were fed daily with dox-containing ESC medium. On d8, cells were detached by using TrypLE™ Select (1×) (Thermo Fisher Scientific, Cat.12563011) and re-seeded at 3×10^5 cells

per well of gelatin coated 6-well dishes for analysis at d18. For *LacZ* and *Ovol1* complementation experiments, cells were assayed at d18 without d8 passage. For continuous culture after d8 sorting, sorted cells were re-seeded at 1×10^5 cells per well of gelatin coated 6-well dishes with 2×10^5 cells of MitomycinC treated MEFs as feeder. Total viable cell counts were performed using a TC20 Automated Cell Counter (Bio-Rad) using trypan blue exclusion.

Whole-Well Fluorescence Microscopy Imaging

Mouse fibroblasts were plated on standard tissue culture 6-well plastic plates (Greiner, Cat. 657160). Images were acquired with a Nikon BioStation CT (Nikon) equipped with GFP and mCherry fluorescence filters and phase contrast using 2× lenses. The single-plane images of each channel were stitched automatically using the automated image analysis software CL-Quant 3.0 (Nikon). Colony count analysis was performed using a custom macro for CL-Quant 3.0. Briefly, iPS colonies were initially identified using phase contrast images which served to generate an outline. Objects identified as colonies were used as regions of interest (ROI) from where the GFP and mCherry fluorescence intensities were measured. The background was subtracted from both the GFP and mCherry fluorescent images. The threshold parameters were set by adding 5 times the standard deviations to the mean intensities of GFP-negative or mCherry-negative colonies in the background subtracted images.

Flow Cytometry and Cell Sorting

For cell surface marker detection, TrypLE™ Select (1×) (Thermo Fisher Scientific, Cat.12563011) was used for cell dissociation. 3×10^5 cells were re-suspended in 100 µL of FACS buffer (PBS contained 2% of FBS) and incubated with primary antibodies and appropriate secondary antibodies on ice for 30 min each. Antibodies used in this study is described in Table S4. The samples were washed with 1 mL of FACS buffer two times after each 30 min incubation with antibodies. The samples were analyzed using a BD LSRFortessa Cell Analyzer (BD Biosciences) with BD FACSDiva software (BD Biosciences). Flow cytometry data were analyzed and generated by FlowJo software. For cell sorting, the cell population was collected on a BD FACSAria II cell sorter (BD Biosciences) following the same process as flow cytometry analysis, using MEF and mESCs as the control of the expression intensity.

Mouse epidermal keratinocytes isolation

Primary keratinocytes were isolated from newborn pups according to (Lichti et al., 2008). Briefly, mice were euthanized at post-natal day 2 by decapitation using scissors. The newborns were surgically de-limbed and subsequently washed in iodine povidone solution, 70% ethanol and finally in distilled water. The skin was removed and placed in PBS^- containing 50 µg/mL Gentamicin (Thermo Fisher Scientific, Cat. 15710-064) for 20 min. The epidermis was dissociated from the dermis by overnight treatment with Dispase II solution 0.5% w/v (Roche, Cat. 165859, 0.5U/mg) at 4^oC. The epidermis was rinsed in PBS^- and cut into small pieces with a scalpel and was further incubated in neat trypsin without EDTA 0.25% (Thermo Fisher Scientific, Cat. 15050065) for 10 min at 37^oC. The keratinocytes were suspended by pipetting up and down, and filtered through 70 µm Nylon mesh filters (BD Falcon). The

trypsin was blocked using one volume of D-KSFM medium containing 10% FCS and supplements (Thermo Fisher Scientific, Cat. 10744-019). After two subsequent washes, the cells were suspended in the same medium and allowed to adhere briefly onto collagen I coated dishes at 4×10^4 cells per cm^2 at 37°C, 5% CO_2. The medium was replaced after 20 min by D-KSFM medium with 10% FCS. The next day the medium was exchanged to D-KSFM medium with supplements containing mEGF (10 µg/mL) and Cholera toxin (10^{-10} M). The keratinocytes were cultured with medium changes every two days and harvested after one week using trypsin.

Microarray Analysis

RNA isolation, data acquisition and data processing were performed as described (Kim et al., 2015). Briefly, total RNA was prepared in parallel from harvested cells using the RNeasy Mini Kit (QIAGEN) according to the manufacturer's instructions. cDNA synthesis and transcriptional amplification were performed using 200 ng of total RNA with the Whole Transcript (WT) Expression Kit (Thermo Fisher Scientific, Ambion/Affymetrix, Cat.4411973). Fragmented and biotin-labeled cDNA targets were hybridized to GeneChip Mouse Gene 1.0 ST arrays (Thermo Fisher Scientific, Affymetrix, Cat.901168) according to the manufacturer's protocols. Hybridized arrays were scanned using an Affymetrix GeneChip Scanner. Probe signal intensities were normalized with the RMA algorithm in GeneSpring. Quality, correlation, and cluster analyses were performed using GeneSpring GX software v13.1 (Agilent Technologies). The averages of two independent experiments were used for the following samples [mESCs, OKMS mCherry$^+$ intermediate reprogramming population (day 8), OK^{+9}MS mCherry$^+$ intermediate reprogramming population (day 8), primary keratinocytes, *Klf4* mCherry$^+$, and *Klf4*$^{+9}$ mCherry$^+$ intermediate reprogramming population (day 6)]. GO term analysis was performed using the NIH DAVID 6.8 Bioinformatics tool (http://david.abcc.ncifcrf.gov/). Microarray data for d6 reprogramming intermediates [LacZ population (described as MEF Mock d2), OKMS mCherry$^+$ intermediate reprogramming population (day 6), OK^{+9}MS mCherry$^+$ intermediate reprogramming population (day 6), *Klf4* mCherry$^+$ intermediate reprogramming population (day 6) and *Klf4*$^{+9}$ mCherry$^+$ intermediate reprogramming population (day 6)] were previously described and deposited in the Gene Expression Omnibus under accession number GSE65468 (Kim et al., 2015). All additional time points, including gene expression analysis for four iPSC clones previously characterized by chimera contribution (Kim et al., 2015) are available under accession number GSE116309.

qRT-PCR analysis

Total RNA were isolated from bulk reprogramming populations at d4 post-dox addition by using the RNeasy Plus Mini Kit (QIAGEN, Cat.74134) according to the manufacturer's instructions. One microgram of extracted total RNA was reverse transcribed with a SuperScript III First Strand Synthesis Kit (Thermo Fisher Scientific, Cat.18080051). qRT-PCR analysis was performed using TB Green Premix Ex Taq II(Tli RNaseH pluse) (Takara, Cat. RR820A) and analyzed with a QuantStudio Real-Time PCR System (Life Technologies). The expression levels of target genes were normalized by the

expression level of GAPDH and calculated relative to the sgRNA(-) condition. Primer pairs for the amplification are shown in Table S4.

Protein Analysis

For Western blot analysis, cells were collected using TrypLE™ Select (1X) and washed two times using PBS before lysis. Total cell lysates were prepared by heating 1×10^6 cells to 70 °C for 10 min in 100 µL NuPAGE LDS Sample Buffer (1×) (Thermo Fisher Scientific, Cat.NP0008) containing DTT at a final concentration of 50 mM. Lysates were resolved on NuPAGE 10% Bis-Tris gels (Thermo Fisher Scientific, Cat.NP0316BOX), and probed using antibodies described in Table S4. Signals were raised using ECL Prime Western Blotting Detection Reagent (GE Healthcare, Cat.RPN2232) and detected on an ImageQuant LAS 4000 imaging system (GE Healthcare).

Immunofluorescence imaging

Cells were fixed with 4%-Paraformaldehyde Phosphate Buffer Solution (Nacalai Tesque, Inc., Cat.09154-85) for 10 min at room temperature, and masked with DPBS(-) containing 100mM glycine for 15 min at room temperature. The cells were blocked with DPBS (-) containing 3% bovine serum albumin (BSA) for 1h at room temperature. The cells were then incubated with primary antibodies described in Table S4 in DPBS (-) containing 3% BSA overnight at 4°C. After being washed in DPBS (-), the cells were stained with secondary antibodies described in Table S4 in DPBS (-) containing 1 µg/mL of DAPI (4',6-diamidino-2-phenylindole) and 3% BSA for 1h at room temperature. After being washed in DPBS (-), samples were analyzed using an IN Cell Analyzer 6000 (GE Healthcare Life Sciences).

Sequencing

Genomic DNA was prepared from harvested cells using the DNeasy Blood & Tissue Kit (Qiagen, Cat. 69506) according to the manufacturer's instructions. sgRNA target sites were amplified by PCR using KAPA HiFi (KAPA Biosystems, Cat.KK2601). Primers are described in Table S4. PCR products were treated with EXOSAP-IT Express (Thermo Fisher Scientific, Cat.78201) prior to sequencing. DNA sequencing was performed using BigDye Terminator v3.1 Cycle Sequencing Kit (Applied Biosystems), purified by ethanol precipitation, and run on a 3130*xl* Genetic Analyzer (Applied Biosystems). Sequence alignments were performed using Snapgene v3.1.4. TIDE analysis of mixed sequences was performed using the online tool at https://tide.nki.nl/. The deletion size window was extended to 25 bp to accommodate larger deletions. The remaining parameters were set to default or allowed to adjust automatically based on the properties of the sequence trace files provided.

EdU staining and detection

EdU staining was conducted using Clik-iT® plus EdU flow cytometry assay kits (Thermo Fisher Scientific, Cat.C10424) according to the manufacturer's instructions. Briefly, cells were cultured with ES medium containing 10 µM EdU for 1 hour at d8 post-dox addition. Cells were harvested using TrypLE™ Select (1×) and fixed by paraformaldehyde contained Click-iT® fixative. Fixed cells were

permeabilized by Click-iT® saponin-based permeabilization and wash reagent, and incorporated EdU was stained with APC using Click-iT® reaction. The samples were analyzed using a BD LSRFortessa Cell Analyzer (BD Biosciences) with BD FACSDiva software (BD Biosciences). Flow cytometry data were analyzed and generated by FlowJo software.

Time-lapse video microscopy

For time-lapse imaging, wild type MEFs were transfected with PB-TAC-OK+9MS IRES mCherry nuclear membrane. The TROP2^{-} and TROP2^{+} reprogramming intermediates were isolated by cell sorting using BD FACSAria III cell sorter (BD Biosciences) on d6. 1×10^4 sorted cells were re-plated in ESC medium with 1 μg/mL of Doxycycline, anti SSEA-1 antibody conjugated with Brilliant Violet421 (BioLegend, Cat.125614, 1:200) and anti Trop2 antibody conjugated with FITC (R&D SYSTEMS, Cat.FAB1122F, 1:100) in μ-slide 8 well (ibidi, Cat.80826) with MEF feeder cells and cultured at 37°C. Each well was overlaid with silicon oil (Sigma-Aldrich, Cat.85419) and gassed on the microscope stage with 5% CO_2 and 5% O_2 using a self-developed continuous gas delivery lid system. Time-lapse imaging microscopy was carried out using Zeiss Axio Observer Z1 microscope (Zeiss) with definitive focus module, SpectraX light engine (Lumencor), 10x Fluar objective (Zeiss), transmitted VIS-LED (Zeiss) and an AxioCamHRm camera (1388x1040 pixel resolution). All hardware was controlled with a VBA module remote controlling Zeiss AxioVision 4.8.2 microscope control software (Filipczyk et al., 2015). Images were acquired on live cells at 50 ms in bright-field, 500 ms for the mCherry channel using an mCherry filter (Cat.F36-508, AHF), 500 ms for the SSEA1 Brilliant Violet421 channel using custom made DAPI filter (Cat, F39-404 for excitation and F47-460 for emission, AHF) and 500 ms for Trop2 FITC channel using EGFP filter (Cat.F46-002, AHF). Cells were imaged continuously at 30 min intervals for 3 days. The first image was acquired 2 hr after sorting. The medium was changed 21h and 43h after starting time-lapse movie. To edit time-lapse movies, tTt was used (Hilsenbeck et al., 2016).

Supplemental References

Filipczyk, A., Marr, C., Hastreiter, S., Feigelman, J., Schwarzfischer, M., Hoppe, P.S., Loeffler, D., Kokkaliaris, K.D., Endele, M., Schauberger, B., *et al.* (2015). Network plasticity of pluripotency transcription factors in embryonic stem cells. Nat Cell Biol *17*, 1235-1246.

Hilsenbeck, O., Schwarzfischer, M., Skylaki, S., Schauberger, B., Hoppe, P.S., Loeffler, D., Kokkaliaris, K.D., Hastreiter, S., Skylaki, E., Filipczyk, A., *et al.* (2016). Software tools for single-cell tracking and quantification of cellular and molecular properties. Nat Biotechnol *34*, 703-706.

Lichti, U., Anders, J., and Yuspa, S.H. (2008). Isolation and short-term culture of primary keratinocytes, hair follicle populations and dermal cells from newborn mice and keratinocytes from adult mice for in vitro analysis and for grafting to immunodeficient mice. Nat Protoc *3*, 799-810.

Ohnishi, K., Semi, K., Yamamoto, T., Shimizu, M., Tanaka, A., Mitsunaga, K., Okita, K., Osafune, K., Arioka, Y., Maeda, T., *et al.* (2014). Premature termination of reprogramming in vivo leads to cancer development through altered epigenetic regulation. Cell *156*, 663-677.

Okita, K., Ichisaka, T., and Yamanaka, S. (2007). Generation of germline-competent induced pluripotent stem cells. Nature *448*, 313-317.

Ran, F.A., Hsu, P.D., Wright, J., Agarwala, V., Scott, D.A., and Zhang, F. (2013). Genome engineering using the CRISPR-Cas9 system. Nat Protoc *8*, 2281-2308.

Woltjen, K., Michael, I.P., Mohseni, P., Desai, R., Mileikovsky, M., Hamalainen, R., Cowling, R., Wang, W., Liu, P., Gertsenstein, M., *et al.* (2009). piggyBac transposition reprograms fibroblasts to induced pluripotent stem cells. Nature *458*, 766-770.
